# An HDAC9-MALAT1-BRG1 complex mediates smooth muscle dysfunction in thoracic aortic aneurysm

**DOI:** 10.1038/s41467-018-03394-7

**Published:** 2018-03-08

**Authors:** Christian L. Lino Cardenas, Chase W. Kessinger, Yisha Cheng, Carolyn MacDonald, Thomas MacGillivray, Brian Ghoshhajra, Luai Huleihel, Saifar Nuri, Ashish S. Yeri, Farouc A. Jaffer, Naftali Kaminski, Patrick Ellinor, Neal L. Weintraub, Rajeev Malhotra, Eric M. Isselbacher, Mark E. Lindsay

**Affiliations:** 1 Thoracic Aortic Center, Massachusetts General Hospital, Harvard Medical School, 55 Fruit Street, Boston, MA 02114 USA; 2 Cardiovascular Research Center, Massachusetts General Hospital, Harvard Medical School, 55 Fruit Street, Boston, MA 02114 USA; 3 Cardiology, Department of Medicine, Massachusetts General Hospital, Harvard Medical School, 55 Fruit Street, Boston, MA 02114 USA; 4 Cardiac Surgery, Department of Surgery, Massachusetts General Hospital, Harvard Medical School, 55 Fruit Street, Boston, MA 02114 USA; 5 Department of Radiology, Massachusetts General Hospital, Harvard Medical School, 55 Fruit Street, Boston, MA 02114 USA; 6 McGowan Institute for Regenerative Medicine and Department of Surgery, University of Pittsburgh, 450 Technology Drive, Pittsburgh, PA 15219 USA; 7 Pulmonary, Critical Care, and Sleep Medicine, Yale University, 300 Cedar Street, TAC-S441D, New Haven, CT 06510 USA; 8 Cardiovascular Genetics Program, Massachusetts General Hospital, Harvard Medical School, 55 Fruit Street, Boston, MA 02114 USA; 9 Augusta University/Medical College of Georgia, 1459 Laney Walker Boulevard, CB3330, Augusta, GA 30912 USA; 10 Pediatric Cardiology, Department of Pediatrics, Massachusetts General Hospital, Harvard Medical School, 55 Fruit Street, Boston, MA 02114 USA

## Abstract

Thoracic aortic aneurysm (TAA) has been associated with mutations affecting members of the TGF-β signaling pathway, or components and regulators of the vascular smooth muscle cell (VSMC) actomyosin cytoskeleton. Although both clinical groups present similar phenotypes, the existence of potential common mechanisms of pathogenesis remain obscure. Here we show that mutations affecting TGF-β signaling and VSMC cytoskeleton both lead to the formation of a ternary complex comprising the histone deacetylase HDAC9, the chromatin-remodeling enzyme BRG1, and the long noncoding RNA MALAT1. The HDAC9–MALAT1–BRG1 complex binds chromatin and represses contractile protein gene expression in association with gain of histone H3-lysine 27 trimethylation modifications. Disruption of *Malat1* or *Hdac9* restores contractile protein expression, improves aortic mural architecture, and inhibits experimental aneurysm growth. Thus, we highlight a shared epigenetic pathway responsible for VSMC dysfunction in both forms of TAA, with potential therapeutic implication for other known HDAC9-associated vascular diseases.

## Abstract (web summary)

Vascular smooth muscle cell (VSMC) dysfunction is a common feature of thoracic aortic aneurysms (TAAs). Here, Lino Cardenas and colleagues show that the formation of a HDAC9-MALAT1-BRG1 complex promotes VSMC dysfunction in TAA by epigenetically altering the expression of key components of the cytoskeleton in VSMCs.

## Introduction

Hereditary thoracic aortic aneurysm (TAA) can be mediated by genetic variation and numerous causal genes have now been associated with the phenotype^1–3^. Several classes of genetic perturbation are directly tied to the function of vascular smooth muscle cells (VSMCs). One group includes genes encoding members of the canonical transforming growth factor-β (TGF-β) signaling cascade (exemplified by the Marfan syndrome and Loeys-Dietz syndrome) and are known collectively as TGF-β vasculopathies (TGFβVs). This class of genetic perturbations includes mutations in the genes *FBN1*, *TGFB2*, *TGFB3*, *TGFBR1*, *TGFBR2*, *SMAD2*, *SMAD3*, and *SKI*^4–10^. The second major group of mutations involves genes encoding component members of the smooth muscle contractile apparatus (Smooth Muscle Contraction Vasculopathies, SMVCs). Gene defects in SMCVs include mutations in the actin-myosin pair: α-smooth muscle actin (encoded by *ACTA2*) and smooth muscle myosin heavy chain (encoded by *MYH11*)^11–13^, as well as genes encoding regulators of the actin-myosin interaction, *PRKG1* and *MYLK*^14,15^. In fact, considering that mutations in genes that define VSMC identity mediate TAA, this phenotype should rightly be considered as a cardinal manifestation of smooth muscle cell dysfunction.

Described human genetic variation indicates that perturbations that either negatively influence canonical TGF-β signaling, or alternatively disrupt vascular smooth muscle cell (VSMC) contraction, result in impaired aortic homeostasis. Despite the functional dissimilarity of these gene groups, patients with TGFβVs and SMCVs display similar anatomic distributions of aortic disease, progressive aortic enlargement, and pathologic elastin fragmentation with loss of markers of contractile smooth muscle cell phenotype^11,16^. On the basis of phenotypic similarity, we reasoned that a shared disease mechanism was discoverable through mutual pathway perturbation and comparison of observed effects.

Herein we show that in the presence of diverse genetic perturbations affecting VSMC cytoskeletal stability, the class IIa histone deacetylase HDAC9 is upregulated, concentrates in the nucleus, and joins into a novel chromatin-remodeling complex with the brahma-related gene 1 protein (BRG1) and the long noncoding RNA (lncRNA) MALAT1. We find the complex associated with repressive chromatin marks at the promoters of key cytoskeletal genes, and inhibition of HDAC9 function relieves disease-associated transcriptional repression. MALAT1 is necessary for targeting HDAC9 to the nucleus and depletion of MALAT1 or HDAC9 reverses deleterious cellular phenotypes and modifies VSMC-dependent pathology including inhibiting experimental aortic aneurysm. We show that HDAC9, in cooperation with BRG1 and MALAT1, mediates a critical epigenetic pathway responsible for VSMC dysfunction. These data offer the first mechanistic link between the two major categories of human genetically triggered aortic aneurysm (TGFβVs and SMCVs), and have therapeutic implications for treatment of not only aortic aneurysm, but also other *HDAC9*-associated human vascular diseases involving VSMC function, including hypertension^17^, intracranial aneurysms^18^, ischemic stroke^19^, and myocardial infarction^20^.

## Results

### TAA-related genetic perturbations upregulate HDAC9

**Fig. 1:**
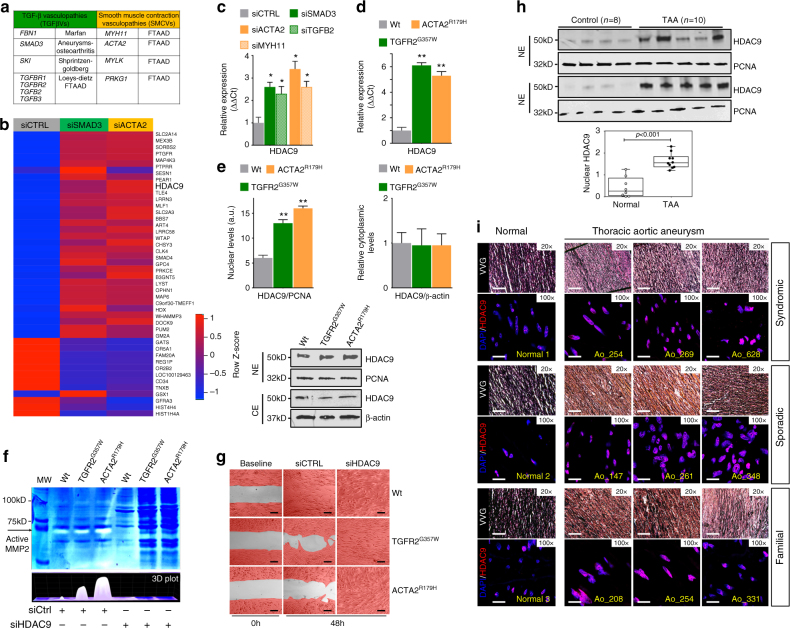
HDAC9 upregulation is associated with TAA-related human genetic variation. **a** *TGFβVs* and *SMCVs* gene groups. **b** Heat map of mRNA expression of commonly dysregulated genes in siSMAD3 (HDAC9 fold change = 1.6) and siACTA2 (HDAC9 fold change = 2.0) treated human VSMCs. The scale bar indicates fold change *Z*-score. **c** QPCR validation of HDAC9 transcript induction in siSMAD3, siTGFB2, siACTA2 and siMYH11 treated cells. Four experimental replicates are plotted. **d** QPCR analysis of HDAC9 transcript in human VSMCs transfected with either empty vector, TGFR2^G357W^, or ACTA2^R179H^ alleles. Four experimental replicates are plotted. **e** Quantitative western blot analysis of HDAC9 distribution in nuclear and cytoplasmic extracts demonstrates increased nuclear localization in the presence of TGFR2^G357W^ or ACTA2^R179H^ alleles. Three experimental replicates are quantified. **f** siHDAC9 treatment of VSMCs reverses MMP activity induced by expression of TGFR2^G357W^ or ACTA2^R179H^ alleles in human VSMCs as measured by in vitro gelatin zymography. **g** siHDAC9 treatment of VSMCs rescues inhibition of migratory activity induced by expression of TGFR2^G357W^ or ACTA2^R179H^ alleles in human VSMCs as assayed by wound healing assay. Serial photomicrographs of wound healing assay at 0 and 48 h are shown, VSMCs are false colored in red. Bar = 200 µM. **h** Quantitative western blot analysis of HDAC9 protein in nuclear extracts from human aortic tissue from TAA patients and non-aneurysmal controls. **i** Immunofluorescence staining demonstrates similar nuclear localization of HDAC9 protein in human TAA samples from diverse forms of TAA etiology. Aortic lumen is rightward facing. (20×, Bar = 50 µM; 100×, Bar = 10 µM). FTAAD, Familial Thoracic Aortic Aneurysms and Dissections; TAA, Thoracic Aortic Aneurysm. Bar graphs are presented as mean with error bars (±S.D.), Student’s *T*-test, *p < 0.05, **p < 0.01 vs. WT. Full-length western blots presented in Supplementary Fig. 8

To investigate shared transcriptional perturbations we created a cellular system using human aortic VSMCs to mimic loss-of-function genetic variation associated with the TAA phenotype. The genes *SMAD3* and *TGFB2* (TGFβVs) as well as *ACTA2* and *MYH11* (SMVCs) fulfilled this criterion and were selected for specific siRNA targeting after proven bioactivity (Fig. 1a and Supplementary Fig. 1a). Of 42542 genes examined, microarray gene expression analysis demonstrated 44 genes dysregulated in the same direction for both siSMAD3-treated and siACTA2-treated cells with a greater than 1.5-fold change (Fig. 1b, Supplementary Fig. 1b). Analysis of the interactome and disease-related network on the 44-dysregulated genes identified *HDAC9*, *SMAD4*, *PTGFR*, and *PRKCE* as the highest scoring genes within the cardiovascular disease category (Supplementary Data 1, Supplementary Fig. 1c). *HDAC9* was validated and selected for further examination due to its implication in diverse forms of human vascular disease^18–20^. Quantitative PCR (qPCR) analysis of siSMAD3, siTGFB2, siACTA2 and siMYH11 treated cells confirmed upregulation of total HDAC9 (Fig. 1c) and individual HDAC9 transcript isoforms (Supplementary Fig. 1d). To extend these observations, we next modeled two aggressive forms of hereditary TAA, by lentiviral expression in human aortic VSMCs, TGFR2^G357W^, a severe Loeys-Dietz syndrome TGFβV^21^ and ACTA2^R179H^, causing a severe SMCV^22^. Dramatic upregulation of HDAC9 was observed in both of these experimental conditions, but not when wild-type versions were overexpressed (Fig. 1d, Supplementary Fig. 1e&f). In the presence of these alleles HDAC9 demonstrates increased nuclear localization (Fig. 1e). Cells from aortic aneurysms have been noted to have migratory abnormalities as well as upregulation of matrix degrading enzymes, such as MMP2 and MMP9^23,24^. Expression of the TGFR2^G357W^ or ACTA2^R179H^ alleles in VSMCs induced increased MMP catalytic activity as well as inhibition of activity in wound healing assays, both of which could be suppressed by silencing of HDAC9 (Fig. 1f, g). Conversely, wound healing assay inhibition could be induced through overexpression of HDAC9/MITR (Supplementary Fig. 1g&h). Similar to cellular models, HDAC9 was found to be grossly upregulated in surgical samples from TAA patients (Fig. 1h). Immunohistochemistry from TAA samples also showed upregulation of HDAC9 in aneurysm tissue from syndromic, sporadic, and familial cases of TAA (Fig. 1i, Supplementary Data 2).

**Fig. 2:**
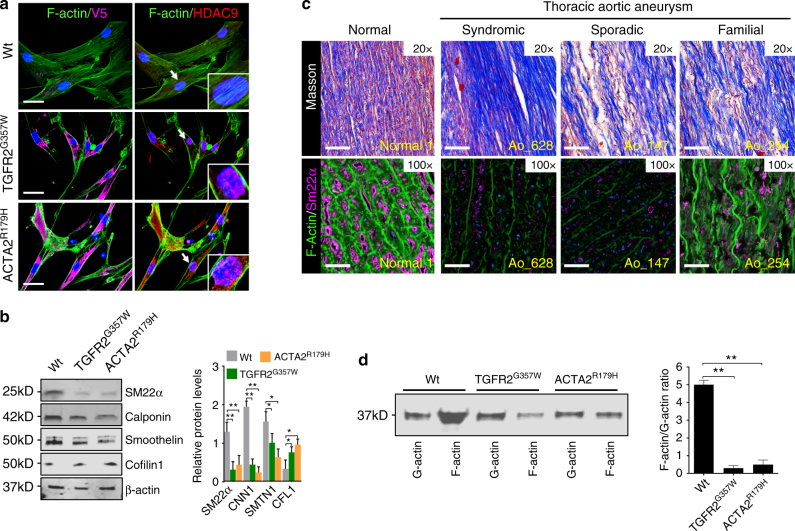
TAA-related human genetic variation destabilizes the actin cytoskeleton. **a** Immunofluorescence of VSMCs transfected with TGFR2^G357W^ or ACTA2^R179H^ alleles (identified by anti-V5 staining, left column), and with anti-HDAC9 stain (red, right column), demonstrate loss of organized actin cytoskeleton structure with anti-F-actin staining, (green, both columns). Bar = 20 µM, arrows indicate focus of 3× zoom inset panel. **b** Western blot demonstrates diminishment of alpha-smooth muscle actin (α-SMA), calponin (CNN1), and smoothelin (SMTN1) expression in with TGFR2^G357W^ or ACTA2^R179H^ expressing cells. Three experimental replicates are quantified. **c** Masson Trichrome staining of human aortic samples (top row) blue color represents collagen deposition, and immunofluorescence of human TAA samples from diverse etiologies (bottom row) demonstrates decreased F-actin staining (green) staining with anti-SM22α (magenta, all columns) when compared to control aortic tissue. Note, F-actin staining is noted between amorphous elastin fiber autofluorescence. (20×, Bar = 50 µM; 63×, Bar = 15 µM). **d** Actin fractionation assay demonstrates decreased F-actin to G-actin ratio in with TGFR2^G357W^ or ACTA2^R179H^ expressing cells. Three experimental replicates are quantified. Bar graphs are presented as mean with error bars (±S.D.), Student’s *T*-test, *p < 0.05, **p < 0.01 vs. WT. Full-length western blots presented in Supplementary Fig. 8

Both the observed deficiency in wound healing assays and induction of MMP activity are reminiscent of phenotypes induced by actin cytoskeletal destabilization^25^. We therefore chose to visualize the actin cytoskeleton in our experimental model. Notably, an apparent loss of F-actin content was a shared feature of TGFR2^G357W^ and ACTA2^R179H^ transduced VSMCs (Fig. 2a). Similarly, expression of several VSMC contractile proteins was decreased (Fig. 2b, Supplementary Fig. 2a). As previously observed in murine aneurysm models^26^ the expression of cofilin-1, an important negative regulator of actin polymerization, was upregulated. A similar decrease in filamentous actin staining and decrement in contractile protein expression (SM22α) is observed in human TAA tissue taken at the time of surgery (Fig. 2c). Consistent with this observation, a decrease in the ratio of F-actin to G-actin is a shared feature of TGFR2^G357W^ and ACTA2^R179H^ transduced VSMCs (Fig. 2d). To determine whether modulation of the actin cytoskeleton could also directly induce the expression and mobilization of HDAC9, we treated VSMCs with the actin toxin latrunculin (1 µM) and the toxin phalloidin (1 µM). Latrunculin (an actin destabilizing agent), induced robust expression as well as rapid nuclear accumulation of HDAC9, whereas phalloidin (an actin stabilizing agent) did not (Supplementary Fig. 2b).

### HDAC9 joins a complex with BRG1 and the lncRNA MALAT1

**Fig. 3:**
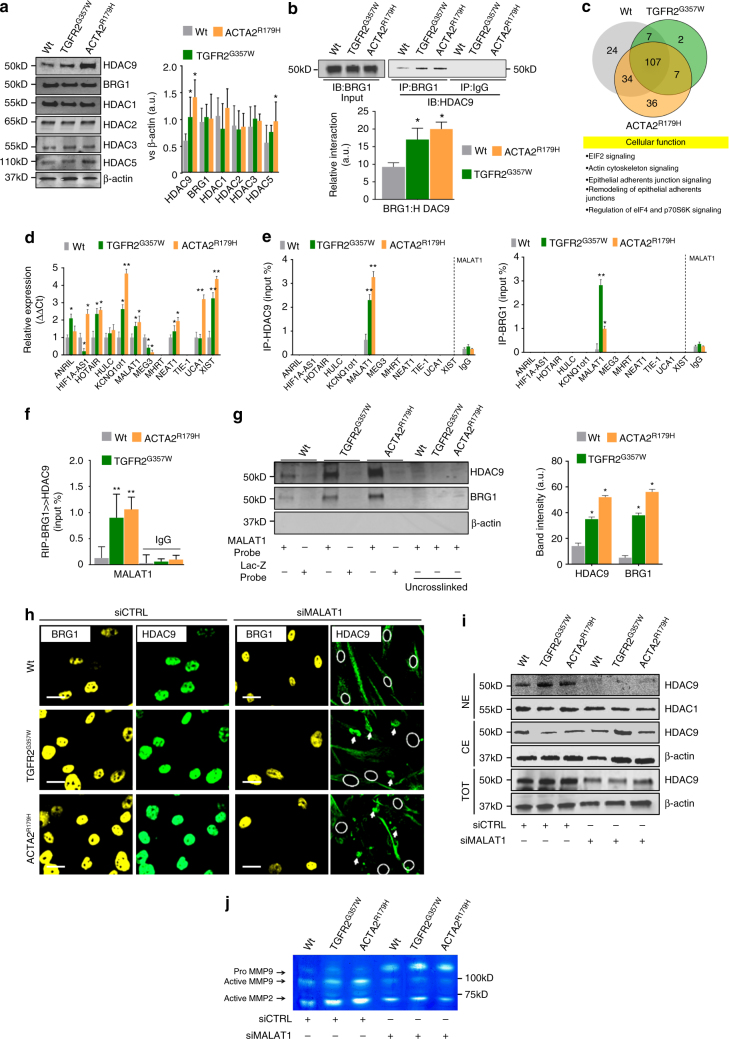
HDAC9 interacts with BRG1 and the lncRNA, MALAT1. **a** Increased expression of HDAC9 protein is noted in TGFR2^G357W^ or ACTA2^R179H^ expressing alleles by western blot when compared to other HDAC isoforms in human VSMCs. Three experimental replicates are quantified. **b** Immunoprecipitation of BRG1 demonstrates HDAC9 binding in TGFR2^G357W^ and ACTA2^R179H^ expressing mutant cells. Quantification of protein levels of three replicate experiments. **c** Top, CLIP-seq analysis of anti-HDAC9 and anti-BRG1 immune complexes illustrated by Venn diagram showing the number of transcripts detected by purification with both antibodies at baseline or during expression of TGFR2^G357W^ or ACTA2^R179H^ alleles. Bottom, cellular function of 107 core transcripts by ingenuity pathway analysis. **d** Quantitative PCR analysis of known cardiovascular lncRNAs during the expression of TGFR2^G357W^ or ACTA2^R179H^ alleles. **e** Quantitative PCR analysis of cardiovascular lncRNAs associated with either anti-HDAC9 or anti-BRG1 immune complexes. Four experimental replicates are quantified. **f** Serial immunoprecipitation of BRG1 and HDAC9 demonstrates the association of the lncRNA MALAT1 with BRG1-HDAC9 immunocomplexes. Quantification of protein levels of three replicate experiments. **g** Western blot analysis of HDAC9 and BRG1 proteins captured through UV crosslinking of bead immobilized biotinylated MALAT1 or LacZ antisense RNA probes. Three replicates are quantified. **h** Immunofluorescence of HDAC9 and BRG1 demonstrate lack of nuclear localization of HDAC9 in siMALAT1-treated cells. Bar = 15 µM, circles indicate nuclei location in siMALAT-treated cells, arrows indicate apparent cytoplasmic HDAC9 aggregates. **i** Semiquantitative western blot analysis of HDAC9 and HDAC1 from nuclear extracts (NE) and cytoplasmic extracts (CE) from control and siMALAT1-treated VSMCs. **j** siMALAT1 treatment of VSMCs decreases TGFR2^G357W^ or ACTA2^R179H^ mediated induction of MMP activity by in vitro gelatin zymography. Bar graphs are presented as mean with error bars (±S.D.), Student’s *T*-test, *p < 0.05, **p < 0.01 vs. WT. Full-length western blots presented in Supplementary Fig. 8

While several other HDAC isoforms demonstrated transcriptional upregulation, only HDAC9 demonstrated clear upregulation at the protein level (Fig. 3a, Supplementary Fig. 3a). To gain insight into cellular events in TGFR2^G357W^ and ACTA2^R179H^ transduced VSMCs, we analyzed HDAC9 via a network analysis (Supplementary Fig. 3b). This revealed the close association of HDAC9 with BRG1 (Brahma-related gene 1), an association noted previously in cardiomyocytes^27^. These data are consistent with previous observations of BRG1 upregulation in TAAs^28^ and involvement of BRG1 in VSMC phenotypic modulation^29,30^. We therefore chose to test for a physical interaction between these proteins. Indeed, HDAC9 strongly associates with BRG1 immunoprecipitates and the interaction is increased in the presence of TAA-associated alleles (Fig. 3b). Given that BRG1 has a known capability to interact with nucleic acids through intrinsic binding activity^31,32^, we hypothesized that the HDAC9–BRG1 complex may associate with nuclear RNAs to regulate chromatin-remodeling events. To address this question we performed genome-wide profiling of HDAC9–BRG1 interactions with RNA using UV crosslinking with high-throughput sequencing analysis (CLIP-seq) from HDAC9 and BRG1 immunoprecipitates in VSMCs. We detected 217 transcripts that associated with HDAC9 and BRG1 at either baseline conditions, or in the presence of the TGFR2^G357W^ or ACTA2^R179H^ alleles, while 107 core transcripts bound the complex under all three conditions (Fig. 3c and Supplementary Data 3). Pathway analysis of the core transcripts revealed them to be thematically involved in EIF2 signaling, remodeling of cell-cell junctions, as well as actin cytoskeletal signaling (Fig. 3c, Supplementary Fig. 3c&d, and Supplementary Data 4). At least two RNAs represented genes directly implicated with aortic disease in humans, *FBN1*^4^ and *LRP1*^33^. In addition to mRNA detection, a single lncRNA was associated with the immunoprecipitates under all experimental conditions, the evolutionarily conserved RNA, MALAT1 (Supplementary Fig. 3e). To test the fidelity of this association, we analyzed HDAC9 and BRG1 immunoprecipitates for binding activity to a panel of known cardiovascular lncRNAs (Fig. 3d). Of these RNAs, only MALAT1 was found to associate specifically with both HDAC9 and BRG1 in CLIP–qPCR assays (Fig. 3e). To test whether MALAT1 bound to HDAC9 or BRG1 individually or within an HDAC9–BRG1 complex, we performed serial immunoprecipitation followed by reverse transcription PCR (RT–PCR) analysis for MALAT1. This assay demonstrated that MALAT1 can be detected associated with HDAC9–BRG1 serial immunoprecipitates (Fig. 3f). Conversely, both BRG1 and HDAC9 are captured in VSMC lysates through the use of MALAT1 antisense probes, but not with a control (lacZ) probe (Fig. 3g). RNA-protein interactions prediction (RPISeq)^34^ confirmed HDAC9 and BRG1 as highest scoring proteins interacting with MALAT1 within *HDAC9* gene association network identified above (Supplementary Fig. 3b, Supplementary Data 5).

We next examined HDAC9 and BRG1 cellular localization during treatment of cells with MALAT1 siRNA. Surprisingly, nuclear localization of HDAC9 is completely dependent on the expression of MALAT1, a result not observed for BRG1 (Fig. 3h, i, Supplementary Fig. 3f). In addition, overall HDAC9 abundance is also reduced in siMALAT1-treated cells (Fig. 3i). Taken together, these results suggest that silencing of MALAT1 expression may inhibit stability of the complex and therefore may be able to prevent cellular phenotypes mediated by the HDAC9–BRG1–MALAT1 complex. Indeed, silencing of MALAT1 decreased the activity of MMP2 and MMP9 in VSMCs expressing TGFR2^G357W^ or ACTA2^R179H^ alleles (Fig. 3j).

### The HDAC9–BRG1–MALAT1 complex associates with gene promoters

**Fig. 4:**
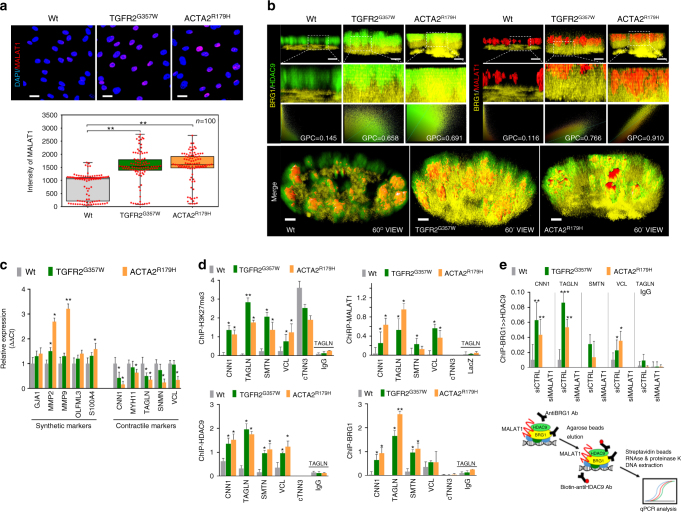
HDAC9–MALAT1–BRG1 complex negatively regulates smooth muscle contractile genes. **a** Fluorescence in situ hybridization (FISH) demonstrates increased levels of MALAT1 in TGFR2^G357W^ or ACTA2^R179H^ expressing cells, (Student’s *T*-test, **p < 0.01). Bar = 15 µM **b** MALAT1, BRG1, and HDAC9 spatially colocalize in TGFR2^G357W^-expressing or ACTA2^R179H^-expressing cells. 3D ultraresolution microscopy showing the intranuclear colocalization of HDAC9 (green), MALAT1(red), and BRG1(yellow) in TGFR2^G357W^ or ACTA2^R179H^ expressing cells. Global Pearson’s colocalization (GPC) of spatial overlap between BRG1-HDAC9 and BRG1-MALAT1 in 100 cells per condition is shown. Bar = 2 µM upper panels, Bar = 1.2 µM lower panels (**c**) Qualitative PCR analysis of molecular markers of synthetic and contractile SMC phenotypes, primarily demonstrate repression of contractile markers in TGFR2^G357W^ or ACTA2^R179H^ expressing VSMCs, (*p < 0.05, **p < 0.01 vs. WT) Four experimental replicates are quantified. **d** ChIP showing increased H3K27me3 marks at the promoters of contractile genes in TGFR2^G357W^ or ACTA2^R179H^ expressing cells, (*p < 0.05 vs. WT). Chromatin immunoprecipitation assays (ChIP) showing increased occupancy of HDAC9 and BRG1 proteins and chromatin isolation by RNA purification (ChiRP) MALAT1 RNA at the promoters of contractile VSMC genes including CNN1 (calponin), TAGLN (Sm22), SMTN (smoothelin), VCL (vinculin), and cTNN3 (troponin) (Student’s *T*-test, *p < 0.05, **p < 0.01 vs. WT). Four experimental replicates are quantified. **e** Serial immunoprecipitation (ReChIP) of BRG1 and HDAC9 demonstrate MALAT1-dependent association of BRG1-HDAC9 immunocomplexes with VSMC contractile promoters. Three replicates are quantified. Bar graphs are presented as mean with error bars (±S.D.), Student’s *T*-test, *p < 0.05, **p < 0.01 vs. WT

MALAT1 is known to localize to the nucleus and shows upregulation in response to TGFR2^G357W^ and ACTA2^R179H^ expression in VSMCs as well as in human TAA tissue (Fig. 4a, Supplementary Fig. 4a). To examine the in vivo spatial relationship between members of this putative complex, we performed intranuclear imaging of BRG1, HDAC9, and MALAT1 in cells expressing TGFR2^G357W^ or ACTA2^R179H^ alleles. In control cells we found close association of HDAC9 with MALAT1, whereas in cells expressing TGFR2^G357W^ or ACTA2^R179H^ alleles, all three members, (HDAC9, BRG1, and MALAT1) show close subnuclear colocalization (Fig. 4b).

**Fig. 5:**
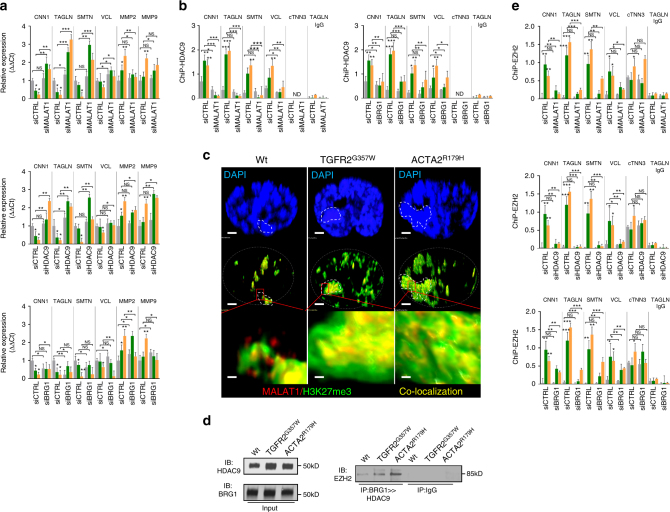
HDAC9–MALAT1–BRG11 is required for EZH2 recruitment to chromatin. **a** Qualitative PCR analysis of synthetic and contractile genes demonstrate HDAC9 and MALAT1-dependant repression in TGFR2^G357W^ or ACTA2^R179H^ expressing VSMCs. **b** HDAC9 chromatin immunoprecipitation assays (ChIP) demonstrates MALAT1 and BRG1-dependant association with VSMC promoters. **c** MALAT1 fluorescence in situ hybridization (FISH) shows colocalization with H3K27me3 marks and areas of dense heterochromatin (dotted line). Bar = 1.2 µM, top and middle row, Bar = 120 nM, bottom row (**d**) Western blot of ectopically expressed EZH2 demonstrating association with sequential BRG1 and HDAC9 immunoprecipitates in TGFR2^G357W^ or ACTA2^R179H^ expressing cells. **e** Chromatin immunoprecipitation assays (ChIP) demonstrate HDAC9, BRG1, and MALAT1-dependant association of EZH2 with VSMC promoters. (Student’s *T*-test, NS not significant, *p < 0.05, **p < 0.01 vs. WT) Bar graphs are presented as mean with error bars (±S.D.), Student’s *T*-test, *p < 0.05, **p < 0.01 vs. WT. Full-length western blots presented in Supplementary Fig. 8

The expression of TGFR2^G357W^ or ACTA2^R179H^ alleles in our model system induced transcriptional events primarily associated with downregulation of contractile proteins (Fig. 4c). Therefore, we analyzed HDAC9–BRG1–MALAT1 complex binding to the proximal promoters of genes known to be repressed in our experimental conditions. Chromatin immunoprecipitation (ChIP and chromatin isolation by RNA precipitation (ChiRP) assays documented increased selective occupancy of HDAC9, BRG1, and MALAT1, but not other HDACs, on the promoters of smoothelin-1 (SMTN1), calponin (CNN1), vinculin (VCL1), and transgelin (SM22α) in the presence of TGFR2^G357W^ and ACTA2^R179H^ aneurysm alleles (Fig. 4d, Supplementary Fig. 4b). This association was closely correlated with increased promoter histone H3K27me3 marks, a well-described marker of transcriptional repression (Fig. 4d). Serial immunoprecipitation (Fig. 2f) and immunofluorescent colocalization (Fig. 4b) demonstrates an HDAC9–BRG1–MALAT1 complex formed in cells expressing TGFR2^G357W^ and ACTA2^R179H^ aneurysm alleles. To determine whether this complex associates with relevant chromatin targets, we performed a ChIP–reChIP experiment. This assay demonstrates a MALAT1-dependant association of the complex with contractile gene promoters (Fig. 4e). In keeping with this observation, MALAT1 and HDAC9 are necessary to mediate repression of contractile gene expression (Fig. 5a) and association of HDAC9 with these promoters was dependent on expression of MALAT1 and BRG1 (Fig. 5b). Unlike MALAT1 and HDAC9, inhibition of BRG1 did not derepress contractile gene transcription, perhaps secondary to the positive role BRG1 plays in VSMC transcription^29,30^.

We next chose to visualize the subnuclear localization of MALAT1 during genetic perturbation. In wild-type nuclei, MALAT1 is closely associated with SC35-containing nuclear speckles as previously described^35^, however in the presence of TGFR2^G357W^ or ACTA2^R179H^ expression, MALAT1 staining was redistributed from euchromatin to regions of heterochromatin indicated by DAPI staining (Supplementary Fig. 5a&b). Consistent with the role HDAC9–BRG1–MALAT1 complex in gene repression in this context we observed colocalization of MALAT1 in subnuclear regions with increased H3K27me3 staining (Fig. 5c). Gene silencing through H3K27me3 chromatin modifications (Fig. 4d) is mediated by polycomb repressive complex 2 (PRC2). Therefore, we investigated the possibility that the HDAC9–BRG1–MALAT1 complex may recruit PRC2 to chromatin to mediate H3K27 methylation. Using a serial co-immunoprecipitaion (Co-IP) approach in lysates from TGFR2^G357W^ or ACTA2^R179H^ expressing VSMCs, we found that EZH2, the catalytic subunit of PRC2, binds to the HDAC9–BRG1–MALAT1 complex (Fig. 5d). Furthermore, ChIP assays demonstrate that MALAT1, BRG1, and HDAC9 are necessary for binding of EZH2 to VSMC promoters (Fig. 5e).

### Targeting HDAC9–BRG1–MALAT1 improves aortic pathology

**Fig. 6:**
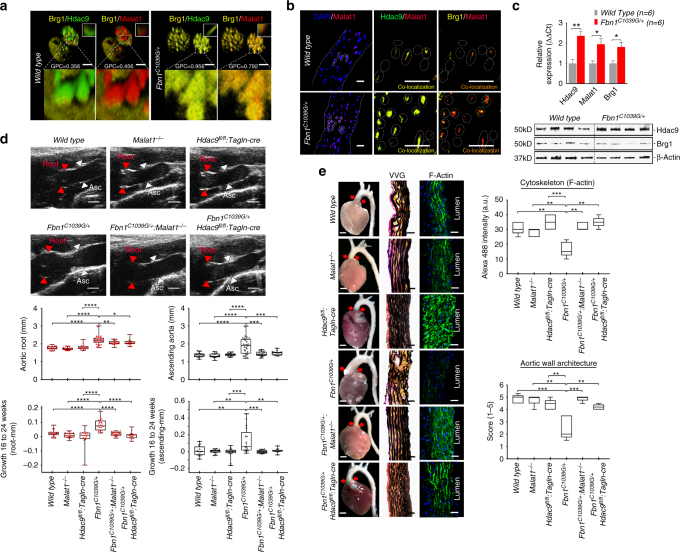
Genetic disruption of Malat1 or Hdac9 improves experimental aortic aneurysm. **a** Explanted VSMCs from *Fbn1*^*C1039G/+*^ mouse aortas demonstrate increased colocalization of Hdac9–Brg1 as well as Malat1–Brg1 when compared to wild-type cells. Global Pearson’s colocalization (GPC) of spatial overlap between Hdac9–Brg1 and Malat1–Brg1 is shown. Bar = 2.5 µM **b** Identification of Hdac9–Malat1–Brg1 complex in wild type and *Fbn1*^*C1039G/+*^ by confocal microscopy of ascending aortic samples. Bar = 40 µM **c** QPCR of *Hdac9*, *Malat1*, and *Brg1* demonstrate upregulation in *Fbn1*^*C1039G/+*^ aortas, bar graphs are presented as mean with error bars (±S.D.) *n* = 6 animals, 6 months of age (**d**) Improved quantitative aortic dimensions in *Fbn1*^*C1039G/+*^*:Malat1*^−/−^ and *Fbn1*^*C1039G/+*^:*Hdac9*^*fl/fl*^*:Tagln-cre* mice when compared to *Fbn1*^*C1039G/+*^ mice. Ultrasound of aortic root (Red) and ascending aortic (Black) quantification of wild-type (*n* = 18), *Malat1*^*−/−*^ (*n *= 18), *Hdac9*^*fl/fl*^*:Tagln-cre* (*n* = 17)*, Fbn1*^*C1039G/+*^ (*n* = 24), *Fbn1*^*C1039G/+*^*:Malat1*^*−/−*^ (*n* = 19), and *Fbn1*^*C1039G/+*^:*Hdac9*^*fl/fl*^*:Tagln-cre* (*n* = 15; One-way ANOVA, *p < 0.05, **p < 0.01, ***p < 0.001). Aortic dimensions at 6 months and aortic growth between 16 and 24 weeks are shown. Representative images of parasternal long axis systolic ultrasound images of the aortic root of 6-month old mice. Red arrows denote aortic root, white arrows denote ascending aorta. Bar = 1 mm (**e**) Malat1 and Hdac9 deficiency rescues anatomic and cytoskeletal defects in *Fbn1*^*C1039G/+*^ aortas. Representative photomicrographs of six month old latex-injected wild-type, *Malat1*^*−/−*^*, Hdac9*^*fl/fl*^*:Tagln-cre, Fbn1*^*C1039G/+*^, *Fbn1*^*C1039G/+*^*:Malat1*^−/−^, and *Fbn1*^*C1039G/+*^:*Hdac9*^*fl/fl*^*:Tagln-cre* hearts and ascending aortas (left column, Bar = 1 mm, middle and right column, Bar = 40 µM). Red arrows indicate the ascending portion of the aorta. Aortas stained with Verhoeff-Van Gieson stain (center panels), and immunofluorescence of F-actin (right panels). The aortic adventitia faces left while the aortic lumen is oriented to the right. Quantification of aortic architecture and F-actin staining, (One-way ANOVA, *p < 0.05, **p < 0.01, ***p < 0.001). Full-length western blots presented in Supplementary Fig. 8

The discovery of an epigenetic pathway induced by genetic perturbations mediating deleterious VSMC phenotypes prompted consideration of modulating this pathway for therapeutic benefit. Initially, we chose to examine for co-association of the members of the HDAC9–BRG1–MALAT1 complex in cells from *Fbn1*^*C1039G/+*^ (Marfan) mice, a validated and widely-used model of hereditary aortic disease. Using confocal microscopy, similar to observations in VSMCs expressing TGFR2^G357W^ or ACTA2^R179H^ alleles, VSMCs explanted from the aortas of *Fbn1*^*C1039G/+*^ mice show increased colocalization of Malat1, Brg1, and Hdac9 when compared to wild-type mice (Fig. 6a). Similarly, we found increased expression of *Hdac9*, *Brg1*, and *Malat1* transcripts and colocalization between members of the complex in *Fbn1*^*C1039G/+*^ mice but not in aortas of wild-type littermates (Fig. 6b, c).

**Fig. 7:**
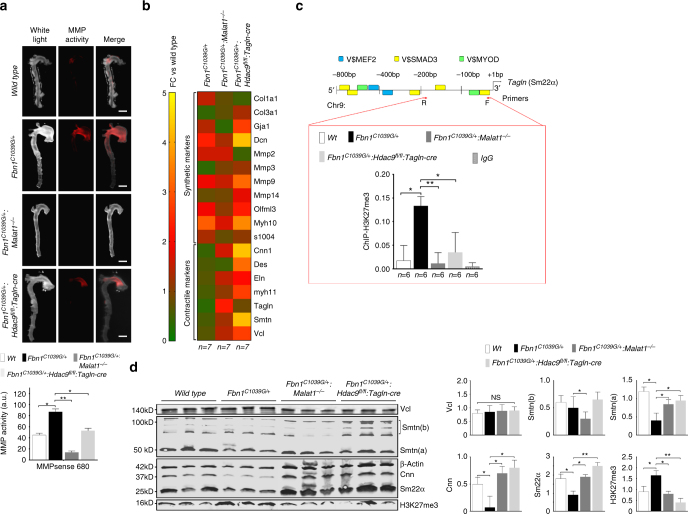
Genetic disruption of Malat1 or Hdac9 restores in vivo contractile protein expression. **a** *Malat1* or *Hdac9* deficiency decreases in vivo aortic MMP activity in *Fbn1*^*C1039G/+*^ mice. Wild-type, *Fbn1*^*C1039G/+*^, and *Fbn1*^*C1039G/+*^*:Malat1*^−/−^, and *Fbn1*^*C1039G/+*^:*Hdac9*^*fl/fl*^*:Tagln-cre* mice are injected with MMP sense 680 in vivo prior to sacrifice and ex vivo imaging. Bar = 1.5 mm. **b** Heat map of contractile and synthetic VSMC markers from ascending aortas of six month old *Fbn1*^*C1039G/+*^, *Fbn1*^*C1039G/+*^*:Malat1*^−/−^, and *Fbn1*^*C1039G/+*^:*Hdac9*^*fl/fl*^*:Tagln-cre* mice. Levels shown by fold change (FC) vs. wild type. (*n* = 7 mice for each genotype). **c** In vivo chromatin immunoprecipitation (ChIP) assays from mouse aortic tissue (*n* = 6 mice for each genotype) using H3K27me3 antibodies at the proximal *Tagln* (Sm22α) promoter. **d** *Malat1* or *Hdac9* deficiency rescues decreased expression of multiple VSMC contractile proteins in *Fbn1*^*C1039G/+*^ mice. Western blot of aortas in wild-type, *Malat1*^−/−^*, Fbn1*^*C1039G/+*^, *Fbn1*^*C1039G/+*^*:Malat1*^−/−^, and *Fbn1*^*C1039G/+*^:*Hdac9*^*fl/fl*^*:Tagln-cre* mice. Quantification of protein levels of three replicate experiments is shown. (Student’s *T*-test, NS not significant, *p < 0.05, **p < 0.01) Bar graphs are presented as mean with error bars (±SD). Full-length western blots presented in Supplementary Fig. 9

We next examined the effect of inhibition of complex formation in *Fbn1*^*C1039G/+*^ (Marfan) mice by crossing them to *Malat1*-deficient (*Malat1*^−/−^) and VSMC targeted *Hdac9*-deficient animals (*Hdac9*^*fl/fl*^*:Tagln-cre*) (Supplementary Fig. 6a-c)^36^. *Fbn1*^*C1039G/+*^:*Malat1*^−/−^ and *Fbn1*^*C1039G/+*^:*Hdac9*^*fl/fl*^*:Tagln-cre* mice demonstrated normal blood pressure (Supplementary Fig. 6d). As assessed by ultrasound, both *Fbn1*^*C1039G/+*^:*Malat1*^−/−^ and *Fbn1*^*C1039G/+*^:*Hdac9*^*fl/fl*^*:Tagln-cre* mice demonstrated smaller ascending aortic dimensions when compared to *Fbn1*^*C1039G/+*^ mice, while the aortic dimensions of *Malat1*^−/−^ and *Hdac9*^*fl/fl*^*:Tagln-cre* mice were indistinguishable from wild-type littermates (Fig. 6d, Supplementary Data 6). The ascending aortas of *Fbn1*^*C1039G/+*^ mice demonstrated elastin fragmentation with VSMC proliferation and disarray^37,38^, a phenotype improved by *Malat1* or *Hdac9* deletion (Fig. 6e). In a corresponding fashion, organized F-actin staining was lost in *Fbn1*^*C1039G/+*^ aortas, but restored by *Malat1* or *Hdac9* deletion (Fig. 6e, Supplementary Fig. 6e). Aortic disease in experimental animals and humans has been associated with increased activation of the TGF-β signaling cascade as assayed by increased phosphorylation of Smad2 and ERK^38,39^. When compared to *Fbn1*^*C1039G/+*^ mice, phosphorylation of both Smad2 and ERK were decreased in the aortas of *Fbn1*^*C1039G/+*^:*Malat1*^−/−^ mice and *Hdac9*^*fl/fl*^*:Tagln-cre* (Supplementary Fig. 6f&g). Previously we demonstrated decreased MMP activation in our cellular models of aneurysm after silencing of the MALAT1 expression (Fig. 2j). Similarly, in vivo MMP activity in the ascending aortas of *Fbn1*^*C1039G/+*^:*Malat1*^−/−^ or *Fbn1*^*C1039G/+*^:*Hdac9*^*fl/fl*^*:Tagln-cre* mice mice was decreased when compared to *Fbn1*^*C1039G/+*^ mice (Fig. 7a). Immunolocalization of MMP2 and MMP9 isoforms within experimental aortas suggests that medial VSMCs are the source of MMP production (Supplementary Fig. 7a&b).

We next undertook qPCR analysis of described markers of VSMC phenotypic state from the aortas of experimental mice. Genetic deletion of *Malat1* or *Hdac9* largely restored “contractile” transcript levels from *Fbn1*^*C1039G/+*^ mice while having no large effect on the expression of “synthetic” markers (Fig. 7b, Supplementary Fig. 6g). Consistent with this, we detected increased H3K27me3 modifications at the *Tagln* promoter in *Fbn1*^*C1039G/+*^ aortas vs. wild-type aortas, while *Malat1* or *Hdac9* deletion restored wild-type levels of this repressive modification (Fig. 7c). Molecular analysis of *Malat1* and *Hdac9*-deficient *Fbn1*^*C1039G/+*^ aortas demonstrated recovery of expression of many extracellullar matrix proteins, including members of the contractile protein apparatus whose levels decreased in *Fbn1*^*C1039G/+*^ mice vs. wild type including restoration of calponin, smoothelin, and Sm22α contractile protein expression (Fig. 7d). Conversely, *Fbn1*^*C1039G/+*^ induced cofilin upregulation was reversed by *Hdac9* or *Malat1* deficiency (Supplementary Fig. 7b).

## Discussion

**Fig. 8:**
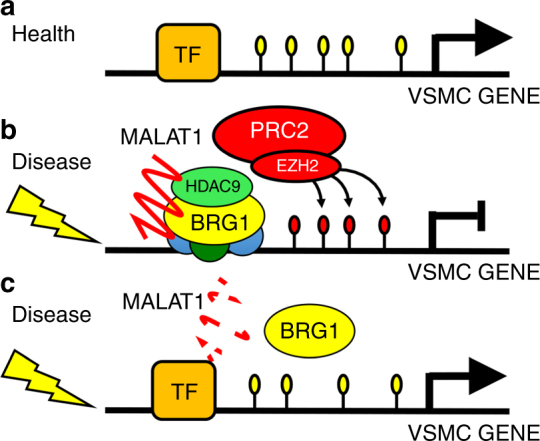
Disease induced transcriptional repression by the HDAC9–BRG1–MALAT1 complex. **a** In the healthy aorta, genes responsible for normal aortic homeostasis are transcribed by multiple transcription factors (TF) such as MEF2, Myocardin, and Myocardin-related transcription factors (MRTFs). **b** Disease processes activate assembly of the HDAC9–BRG1–MALAT1 complex. The promoters of target genes are silenced through the action of the complex and the acquisition of histone 3 lysine 27 trimethylation through polycomb repressive complex 2 (PRC2). **c** Reduced dosage of MALAT1 and/or HDAC9 causes destabilization of the complex and allows for expression of silenced gene loci and reversal of deleterious phenotypes

Although often explored in developmental contexts, epigenetic reprogramming is an emerging theme in homeostatic control of differentiated cell types. Large-scale transcriptional changes driving phenotypic events often involve the coordinated action of nucleoprotein complexes that have adaptable function depending on cellular and tissue type requirements. In this study, we demonstrate that the HDAC9–BRG1–MALAT1 complex represents one such complex involved in pathologic reprogramming of VSMCs (Fig. 8).

Human sequence variants at the *HDAC9* locus have been associated with vascular diseases such as large vessel ischemic stroke^19^, myocardial infarction^20^, intracranial aneurysm^18^, and aortic calcification (R. Malhotra, personal communication). HDAC9 appears to be the disease relevant gene at this locus based on expression studies^40^, arguing strongly for the importance of this factor in vascular cellular performance. However, the mechanism by which HDAC9 mediates vascular disease is not clear. Experimental data in rodents implicates augmented HDAC9 activity in multiple tissue types including adipocytes and macrophage. For instance, in experimental atherosclerotic disease HDAC9 has been shown to play a directive role in the modulation of cholesterol efflux and the generation of alternatively activated macrophages^41^. In the adipocytic lineage, *Hdac9* downregulation is required for differentiation of preadipocytes into mature adipocytes, and knockdown of *Hdac9* prevents metabolic dysregulation in high-fat feeding^42,43^. However, genetically triggered TAA is a noninflammatory and nonatherogenic process, and human genetic perturbations that cause TAA clearly implicate dysfunction of VSMCs^44^. The data presented here indicate a novel VSMC-specific function for HDAC9 from those previously implicated in cardiovascular disease. Indeed, considering its wide and variable expression within the vascular system, mechanistic interpretation of human *HDAC9* genetic associations that implicate a single cell type or even a single pathologic process may be simplistic.

Genetic mutations that disrupt cytoskeletal architecture induce both HDAC9 expression and the formation of the HDAC9–BRG1–MALAT1 complex. We speculate that disruption of the actin cytoskeleton mediates the lack of wound healing function with increased MMP activity seen in our assays (Fig. 1f, g, Supplementary Fig. 1a), uncommon behavior for VSMCs where increased mobility is typically coupled with MMP upregulation. The mechanism by which actin destabilization signals formation of the nuclear complex is unclear. One candidate would be an increased concentration of monomeric beta-actin itself, which has been repeatedly found bound within BRG1-containing complexes^45^. However, we could not detect direct beta-actin binding to the HDAC9–BRG1–MALAT1 complex, and therefore such a mechanism remains speculative. Nuclear localization of HDAC9 appears to be dependent on MALAT1 expression. We hypothesize that MALAT1 may either directly regulate HDAC9 phosphorylation or more likely exclude binding of HDAC9 to 14-3-3 proteins, known to be involved in cytoplasmic retention of class II HDACs^46^. It is formally possible that increased nuclear concentrations of HDAC9 and MALAT1 are sufficient to initiate formation of the HDAC9–BRG1–MALAT1 complex, indeed the expression of these two factors is coreguated as evidenced by decreased HDAC9 levels after suppression of MALAT1 through siRNA silencing (Fig. 3i). While concentration dependent assembly may dictate complex formation, it seems probable that signal-mediated assembly mechanisms may also be elucidated. We do observe the complex associating with the promoters of cytoskeletal genes and with repressive chromatin marks within nuclear regions with repressive character in response to actin destabilization. These findings are consistent with the previous implication of HDAC9 in the repressive control of transcriptional programs^47^. It should be noted that although the majority of studies have documented downregulation of contractile protein elements in TAA^48–51^, at least one study has seen upregulation^52^, demonstrating that protein expression control mechanisms within TAA subtypes may be more diverse than suspected. The catalytic domain of HDAC9 has been shown to be dispensable for its role in adipogenesis, and indeed we that found abnormal VSMC phenotypes could be induced through overexpression of MITR, an HDAC9 isoform lacking its catalytic domain. Therefore, the role of HDAC9 in the complex may primarily be the recruitment of other chromatin modifying factors, such as PRC2, to sequence-specific chromatin binding sites through interaction with MEF2 or other transcription factors^53,54^.

The other member of the complex, MALAT1, is a well known constituent of SC35-containing nuclear speckles, nuclear domains rich in pre-mRNA splicing factors^55^. Indeed, as seen in most cell types, colocalization of MALAT1 with SC35 and euchromatin are noted in VSMCs at rest (Supplementary Fig. 5a). However, in the presence of aneurysm-associated alleles MALAT1 is more closely associated with heterochromatin and regions of dense H3K27 trimethylation (Fig. 5c). These data imply a repressive rather than facilitative role for MALAT1 in gene expression in this context, in contrast to observations in healthy cells^56^. This type of functionality has however been previously observed in MALAT1 biology, in fact, the 3’ end of MALAT1 has been described to bind directly to and recruit EZH2, the catalytic subunit of the PRC2 complex to mediate silencing of target genes^57,58^. Deletion of MALAT1 or treatment with antisense oligonucleotides targeting MALAT1 has been shown to promote differentiation of mammary tumors and prevent metastasis in experimental mice^59^. Gene expression changes in these cells were noted to be diverse and both activation and repression of gene sets were noted in RNA-seq experiments^59^. Although we have studied repressive events in this study, it is quite plausible that activating transcriptional events and modulation of splicing events mediate equally important phenotypes and inhibition of these events by MALAT1 deficiency may motivate improvements in VSMC performance. In fact, considering the commonalities observed between cancer cells and synthetic VSMCs (increased MMP expression, cytoskeletal instability, etc.), the ability of MALAT1 depletion to induce a differentiated phenotype may prove a generalized strategy to improve cellular performance in multiple conditions associated with cellular dedifferentiation.

Recently, an HDAC-BRG1 complex was implicated in stress responsive gene repression in experimental cardiac hypertrophy, possibly an analogous complex operating within cardiomyocytes^27,31^. Indeed, we demonstrated contractile promoter occupancy of other HDAC isoforms, (HDAC1, HDAC2, and HDAC3) under our experimental conditions (Supplementary Fig. 4b), albeit not consistently. In cardiomyocytes, the HDAC9–BRG1 complex was shown to be inhibited by the lincRNA *Mhrt*, which prevents its association with chromatin by competitive binding at the helicase domain of BRG1. In contrast, the lincRNA MANTIS has been shown to facilitate BRG1 function in endothelial lineages^60,61^. In our study, the lncRNA MALAT1 is required for proper association between BRG1 and HDAC9, and depletion of MALAT1 inhibits both stable formation of the complex and the nuclear localization of HDAC9. These data illustrate how regulatory RNAs can work as both positive and negative modulators of transcription in differentiated cell types. As expected from its location within the *Myh7* locus, *Mhrt* was not

expressed in VSMCs (Fig. 3d) and it remains to be seen if smooth muscle lineages have evolved a specific lincRNA to antagonize BRG1-chromatin interactions.

*HDAC9* has been associated with multiple human vascular diseases including hypertension, stroke^19^, aneurysm^18^, and myocardial infarction^20^ amongst others. The mechanistic underpinnings of this genetic association is unclear, however VSMC dysfunction is believed to be fundamentally related to the pathogenesis of each of these vascular disorders. The elucidation of a novel HDAC9-containing complex mediating transcriptional dysregulation in VMSCs may open new avenues for therapeutic intervention for these important human diseases.

## Methods

### Aortic Samples

Aortic samples were collected from patients undergoing cardiac surgery at the Massachusetts General Hospital (MGH). Demographics of thoracic aortic aneurysm patients are listed in Supplementary Table 2. Control aortic tissue was obtained from patients undergoing orthotopic cardiac transplant. IRB permissions do not allow for demographic information from discarded tissue to be collected or stored. All tissue samples were collected in compliance with the Partners Healthcare Institutional Review Board (IRB) requiring written informed consent.

### Aortic smooth muscle cell lines

Primary human aortic smooth muscle cells (VSMC) from healthy donors were purchased from Cell Applications Inc. (354K-05a), California, USA. In order to preserve cell identity all experiments were carried out at passages 1–5^62^. Primary VSMC from wild-type and *Hdac9*^−/−^ mice were isolated from the proximal ascending section of aortas by standard explant protocol. Smooth muscle cell identity was assessed by immunofluorescence staining of contractile markers including Sm22α (Abcam, ab14106), Calponin1 (Abcam, ab46794), Smoothelin1 (Santa Cruz, sc-73042), and Vinculin1 (Abcam, ab18058).

### siRNA inhibition, wound healing and microarrays

Human aortic smooth muscle cells (HAoSMC) were acquired from Cell applications, Inc. (354K-05a) and seeded into silicone inserts plates with a defined cell-free gap (Ibidi-labware) and transfected 24hrs with specific siRNA against *SMAD3*, *TGFB2*, *ACTA2* and *MYH11*. After 24 h silicone inserts were removed to generate the initial wound. No antiproliferative agents were added to the media, therefore wound healing in our assay reflects the combined activity of migration and proliferation. The wound healing/closure was evaluated 24 and 48hrs later. Validation of the gene silencing was performed by qRT–PCR and immunoblotting for each targeted gene (Supplementary Fig. 1g). Total RNA was extracted from HAoSMC transfected with siRNA-negative control, siSMAD3 and siACTA2 using miRNeasy kit (Qiagen) following the manufacturer’s protocol. RNA samples were used to hybridize Agilent gene expression microarrays (v2. mRNA microarrays, Agilent). Briefly, 100 ng of total RNA was used as the starting template for cDNA synthesis. This cDNA was used as a template to synthesize Cy3-labeled cRNA that was hybridized on 8 × 60 K high density SurePrint G3 gene expression human Agilent microarrays at 65 °C for 17 h. All arrays were normalized by cyclic-LOESS and significance analysis of microarrays (SAM) was applied for statistical analysis of the differentially expressed genes setting a 5% false discovery rate as a cutoff of statistical significance. 44 genes were found to be dysregulated in the same direction for both siRNA conditions compared with the control group. Fourteen genes were randomly chosen to be validated by qRT–PCR. RNA samples were used to run qRT–PCR on SYBR green system (Applied Biosystem, Foster city, CA). Results were analyzed by the ddCT method and GAPDH (encoding glyceraldehyde-3-phosphate dehydrogenase) was used as a housekeeping gene. Fold change were calculated by taking the average over all the control samples as the baseline.

### Network analysis

Ingenuity Pathway Analysis (IPA, Ingenuity Systems) software was used to identify potential networks and canonical pathways associated with the commonly dysregulated genes; all 44 differentially expressed genes were included in the analysis. The predicted activation state and activation *Z*-score are based on the direction of fold change values for those genes in the input data set for which an experimentally observed causal relationship has been established.

### Production of aneurysm alleles in vitro

Human wild-type cDNAs of *TGFBR2* and *ACTA2* were separately cloned into a lentivirus plasmid containing a V5 epitope tag and an Emerald Green Fluorescent Protein (EmGFP). Point mutations for TGFR2^G357W^ and ACTA2^R179H^ were introduced with primers (Supplementary Data 7) for site-directed mutagenesis by PCR designed basically according to the manufacture (QuikChange™ Mutagenesis kit; Agilent Technologies, CA). Viruses were produced using the ViraPower lentiviral expression system (Invitrogen) according to the manufacturer’s protocol. After 96 h of viral infection, cells expressing wild-type or mutant proteins were detected using fluorescent microscopy (FITC) or Blasticidin selection. RNA and protein levels were analyzed 48hrs after viral expression. Cell viability was assessed using CellTiter-Glo® 2.0 Assay (Promega) according to the manufacturer’s instruction. Determination of proliferation profile in wild-type or TGFR2^G357W^ and ACTA2^R179H^ mutant cells was measured by immunofluorescence detection of Ki67 and PCNA.

### Actin cytoskeleton disruption assay

Human VSMCs were treated with Phalloidin (1 µM) or Latrunculin A (1 µM) for 40 min before preparation of nuclear protein extracts and analyzed by SDS–PAGE/western blot. For living cell microscopy, human HDAC9/MITR cDNA lacking the HDAC catalytic domain were cloned into a lentivirus plasmid encoding the tdTomato fluorescent protein tag from Takara Bio USA, company (pLVX-tdTomato-N1). Then human VSMCs were transduced with virus particles overexpressing tdTomato-HDAC9 followed by treatment with Latrunculin A (1–3 µM) for 40 min using Leica TCS SP8 microscopy station.

### Immunoblotting and immunoprecipitation

All uncropped western blots are presented in Supplementary Figs. 8&9. All antibodies used in the study are summarized in Supplementary Data 7. Nuclear and cytoplasmic protein lysates were prepared using NE-PER Kit (Pierce, Rockford, IL, USA) and supplemented with 1× of protease inhibitor cocktail (Roche) according to the manufacturer’s instruction. For detection of nuclear proteins 20 µg of total nuclear extracts were mixed with denaturing buffer (1× Laemmli loading buffer with 10% of β-mercaptoethanol) and analyzed by SDS–PAGE/western blot. Separated proteins were transferred onto a nitrocellulose membrane using the iBlot transfer system (Novex, ThermoFisher, USA). For detection of cytoplasmic proteins 15 µg of cytoplasmic extracts were mixed with denaturing buffer as described above. In general, primary antibodies were used at concentration of 1:100 and secondary at concentration of 1:10000, although specific concentrations are listed in Supplementary Data 7. For co-immunoprecipitation assays 40 µg of nuclear extracts were incubated with primary antibody (5 µg) overnight at 4 C followed by co-immunoprecipitation using Pierce Co-IP kit (Thermo Scientific) according to the manufacturer’s instructions. Then lysates were incubated with A/G magnetic beads for 2hrs at 4 C and washed three times with IP buffer (Santa Cruz biotechnologies, USA). Eluate was boiled in denaturing buffer for 10 min at 100 C and analyzed by SDS–PAGE/western blot. For sequential Co-IP 200 µg of nuclear extracts were supplemented with RNAse inhibitor (1:100), protease inhibitor cocktail, phenylmethylsulfonyl fluoride (1 mM, PMSF) and treated with turbo DNAse I for 30 min at 37 C. Then lysates were incubated with 5 µg of BRG1 (Abcam, ab110641) antibody overnight at 4 C on a rotator. Next samples were incubated with A/G agarose beads for 2 h at 4 C on a rotator followed by 3× washes with lysis buffer using centrifugation to pull-down agarose beads. Then samples were resuspended in 1 mL of dilution buffer. Meanwhile 5 µg of HDAC9 (Abcam, ab59718) antibody per sample was biotinylated using Biotin (Type B) Fast Conjugation Kit (Abcam, ab201796). Resuspended samples were then incubated with 5 µg of Biotinylated-HDAC9 antibody for 4 h at 4 C on a rotator followed by incubation with magnetic streptavidin beads for 2 h at 4 C on a rotator. Beads were 3× washed and boiled in denaturing buffer for 10 min at 100 C and analyzed by SDS–PAGE/western blot. For F-actin/G-actin ratio determination cells were treated with Lysis and F-actin stabilization buffer from G-Actin/F-actin. In Vivo Assay Biochem Kit and according to the manufacturer’s instruction (Cytoskeleton, Inc., USA). Briefly 30 µg of stabilized lysates were first centrifuge at 350 × g for 5 min to remove cellular debris. Then supernatants were ultra centrifuged at 100000×*g* for 1 h. After centrifugation G-actin was collected from the supernatants and F-actin was collected from the pellets. Equal volumes (25 µL) from each fraction were analyzed by SDS–PAGE/western blot analysis as above. For protein analysis in animals, the ascending sections of the aorta were used to prepare total protein extracts using T-PER tissue protein extraction buffer (Thermo scientific) followed by 2 × 5 min of mechanic homogenization using TissueLyser LT (Qiagen, USA). 20 µg of total protein per sample were used. The Odyssey infrared western system was used to detect target proteins. Band intensity was quantified using ImageJ software. Uncropped scans of all the blots presented in the manuscript are shown in Supplementary Fig. 5a&b.

### Protein precipitation by MALAT1 isolation

10 million cells of either wild type or aneurysm mutant cells were UV cross-linked using 2000 µJoules/cm^2^ followed by extraction of nuclear protein using NE-PER Kit (Pierce, Rockford, IL, USA) and as previously described^63^. Briefly 50 µg of UV cross-linked nuclear lysates were supplemented with RNAse inhibitor (1:100), protease inhibitor cocktail (1 × ), phenylmethylsulfonyl fluoride (1 mM, PMSF) and treated with turbo DNAse I (Ambion, ThermoFisher, USA) for 30 min at 37 C. Nuclear lysates were mixed with biotinylated DNA probes targeting MALAT1 or LacZ and incubated at 55 C for 3 h. before overnight incubation with yeast RNA-blocked streptavidin magnetic beads at 37 C. Beads were washed three times and retreated with turbo DNAse I for 10 min at 37 C. Beads were washed three more times and incubated with 20 µl of denaturing buffer at 100 C for 10 min followed by SDS–PAGE/western blot analysis.

### MMP activity

For in vitro MMP activity, VSMCs expressing wild type or TGFR2^G357W^ and ACTA2^R179H^ were co-transfected with 30 nM of siCTRL or siHDAC9 for 48hrs as described above. After 24hrs of growing medium 30 µg of total protein were prepared from siCTRL and siHDAC9 treated cells to measure the MMP activity by gelatin zymography^59^. 3D plot was generated using ImageJ software. For in vivo MMP activity assay, mice were tail vein injected with 600uL of MMPSense 750 FAST, a near-infrared fluorescence sensor for MMP2 and MMP9 activity, (PerkinElmer, USA). Mice were killed 24 h post injection and aortas were dissected and analyzed using a Kodak image station 4000MM Pro for macroscopic fluorescence reflectance molecular imaging^64^.

### Sequential FISH and immunofluorescence microscopy

Stellaris® FISH Probes recognizing human MALAT1 (SMF-2035-1) or mouse Malat1 (SMF-3008-1) and labeled with Quasar® 570-labeled oligos (Biosearch Technologies, Inc., Petaluma, CA) were hybridized to VSMCs or tissue samples, followed by incubation with primary and secondary antibodies following the manufacturer’s instructions available online at www.biosearchtech.com/stellarisprotocols. Imaging and analysis were performed using Volocity 5.2 software. Three-dimensional and quantitative fluorescence co-localization analysis were performed as described previously^65^. Two-dimensional and white light images were analyzed using ImageJ software.

### ChIP–qPCR, ChIP–reChIP and ChiRP

For ChIP–qPCR 10 million cells were used from wild type, TGFR2^G357W^ and ACTA2^R179H^ and were fixed with 1% of formaldehyde at 37 C for 20 min, quenched with 125 mM glycine for 5 min (RT) and protein lysates were prepared using EpiTect ChIP kit according to the manufacturer’s instructions (Qiagen, USA). For ChIP–qPCR from mouse aortas 30 mg of fresh tissue corresponding to the ascending region was fixed with 1% of formaldehyde at 37 C for 30 min, quenched with 125 mM glycine for 5 min (RT) and protein lysates were prepared as described above. ChIP-ed DNA was used to analyze the promoter occupancy of contractile genes by qPCR. For ChIP–reChIP^66^, wild type, TGFR2^G357W^ and ACTA2^R179H^ were transfected with siCTRL or siMALAT1 as described above. Then 50 million cells from either siCTRL or siMALAT1-treated cells were fixed with 1% of formaldehyde at 37 C for 20 min quenched with 125 mM glycine for 5 min (RT). Then protein lysates were supplemented with 1× of protease inhibitor cocktail (Roche), RNAse inhibitor (1:50) and 1 mM PMSF and incubated on ice for 15 min. Next lysates were sonicated to shear chromatin to an average length of 500-1500 bp followed by centrifugation for 10 min at max speed. Supernatants were collected in 2 mL tube containing 6 µg of BRG1 (Abcam, ab110641) antibody and 1 mL of lysis buffer supplemented with 1× of protease inhibitor cocktail (Roche), RNAse inhibitor (1:50) and 1 mM PMSF and incubated overnight (14 h) at 4 C. Next samples were incubated with A/G agarose beads for 1 h at 4 C on a rotator followed by 3× washes with lyses buffer using centrifugation to pull-down agarose beads. Then samples were resuspended in 1 mL of dilution buffer as described previously. Meanwhile 6 µg of HDAC9 (Abcam, ab59718) antibody per sample was biotinylated using Biotin (Type B) Fast Conjugation Kit (Abcam, ab201796). Resuspended samples were then incubated with 6 µg of Biotinylated-HDAC9 antibody for 4 h at 4 C on a rotator followed by incubation with magnetic streptavidin beads for 1 h at 4 C on a rotator. Beads were 3× washed and ChIP-ed DNA was prepared using EpiTect ChIP kit as described above. We carried out ChIRP assays as described by Chu and collaborators, 2012^67^. Briefly 50 million cells were used from wild type, TGFR2^G357W^ and ACTA2^R179H^ and cross-linked with 1% glutaraldehyde for 20 min at RT. Then cells were harvest in lysis buffer supplemented with 1× of protease inhibitor cocktail (Roche), RNAse inhibitor (1:50) and 1 mM PMSF followed by sonication as described previously. Next resuspended chromatin was incubated with biotinylated probes recognizing MALAT1 or LacZ (negative control) transcripts for 4hrs on a rotator followed by incubation with magnetic streptavidin beads (100 µL) for 1 h at RT. RNA and DNA isolations were performed as described^67^. QPCR was used to analysis signals in input and immunoprecipitates. The percentage of immunoprecipitates signals was calculated over the input signals. Experiments were performed in triplicates, with independent samples. Promoter locus of cardiac troponin 3 (cTNN3) was used as negative control in the ChIP experiments. Primers used for the qPCR are listed in Supplementary Data 7.

### CLIP-seq

For CLIP-seq 100 million cells were UV cross-linked using 2000 µJoules/cm2 followed by extraction of nuclear proteins. UV cross-linked lysates from wild-type, TGFR2^G357W^ and ACTA2^R179H^ cells were pre-supplemented with RNAse inhibitor (1:50), protease inhibitor cocktail, phenylmethylsulfonyl fluoride (1 mM, PMSF) and treated with turbo DNAse I for 30 min at 37 C. Lysates were then incubated with 5 µg of HDAC9 or BRG1 antibodies at 4 C overnight. RNA extraction from HDAC9 and BRG1 pull downs were performed using miRNeasy kit (Qiagen, USA) according to the manufacturer’s instruction. RNA sequencing for human mRNA and lncRNAs were performed using Ribo-Zero™ Magnetic Kit and RnaseH is used to remove rRNA, remained RNA is then retrieved by ethonal precipitation. The retrieved RNA is fragmented using Ambion Fragmentation Solution. And according to Illumina solexa transcriptome sequencing protocol, random hexamer-primer was used to synthesize the first-strand cDNA. The second-strand cDNA is synthesized using buffer, dNTPs, RNaseH and DNA polymerase I. Fragments are purified with QiaQuick PCR extraction kit and resolved with EB buffer for end reparation and adding A at 3’ end. After that, the fragments are ligated with sequencing adaptors. UNG is added before synthesizing the second cDNA strand. After the second strand degraded, dUTP is then converted to dTTP. Y-adaptor is added afterwards. Suitable fragments are selected as templates according to the agarose gel electrophoresis results for PCR amplification. Generated libraries were sequenced using Illumina HiSeq™ technology. CLIP-seq reads were aligned to human UCSC h19 using standard Illumina library-type. Cufflinks 2.0.2 was run on merged bam files to assemble of transcripts and estimation of FPKM values. FPKM ≤ 1.0 cutoff was used as detection threshold to generate the list of genes. For coverage of MALAT1 transcript the fastqs files were aligned to the human genome (hg19) using STAR^68^. Bedtools CoverageBed (v2.26 http://bedtools.readthedocs.io/en/latest/content/tools/coverage.html) is used for each of the sorted (by chromosome position) alignment bam files to obtain the red coverage for each base in the MALAT1 gene region. The BED file for MALAT1 was obtained from ENSEMBL 75 for the canonical transcript that has a length of 8708 bases. The read coverage for each sample is then normalized to reads per million mapped to the human genome (RPM) by considering only the reads that map uniquely to the human genome respectively. Genomic data from CLIP-seq experiments was deposited to *GEO* under the access number GSE41607.

### Mice

All mice were cared for under strict compliance with the Partners Institutional Animal Care and Use Committee (IACUC), regulated by the United States Public Health Service (USPHS) and the United States Department of Agriculture (USDA). *Malat1* knockout mice were kindly provided by Dr. David Spector^36^. *Hdac9* knockout aortic VSMCs were derived from mice kindly provided by Dr. Eric Olson^47^. *Hdac9*^*flox/flox*^ were generated and kindly provided by Dr. Neal Weintraub. Tagln-cre mice were acquired from Jackson laboratories (B6.Cg-Tg(Tagln-cre)1Her/J, # 017491)^69^ Marfan (*Fbn1*^*C1039G/+*^) mice were acquired from Jackson laboratories (B6.129-*Fbn1*^*tm1Hcd/J*^, #01885)^37^. For tissue analysis, animals were euthanized through inhalational isoflurane (Sigma, St, Louis, MO) prior to tissue collection. Age and sex of all animals used in the study are described in Supplementary Data 6. All tissue experiments were performed after sacrifice at 6 months of age unless described differently. All experiments were performed on male and female animals at a 1:1 ratio.

### Ultrasounds

Nair hair removal cream was used on all mice the day prior to ultrasounds. All ultrasounds were performed on awake, unsedated mice using the Visualsonics Vevo660 imaging system and a 30 MHz transducer. The aorta was imaged using a standard parasternal long axis view. Dimensions from each animal represent averages of measurements made on still frames in systole of the maximal internal diameter of the aortic valve annulus, aortic sinuses, sinotubular junction, or ascending aorta by a cardiologist blinded to genotype. On the basis of historic controls, the mean diameter of the wild-type aorta at 6 months of life is 1.75 + /- 0.2 mm while in our disease model (*Fbn1*^*C1039G/+*^) the mean diameter is 2.2 ± 0.3 mm. We calculated a need for ~10 animals per group at a power (1-β) of 0.8 and a Type I error rate (*α*) of 5% to detect a 0.34 mm (57%) mean improvement (vs. 2.2 mm mean) improvement in aneurysm diameter.

### Histology

Latex was injected into the left ventricular apex under low pressure until it was visible in the femoral artery. Animals were then fixed in Formalin (10%) for 24 h before transfer to 70% ethanol for dissection and storage. Aortas were then removed from the animals or dissected in situ for photography prior to paraffinization and sectioning (7 µM). Slides were produced for tissue staining or stained with standard stains including Elastin (Verhoeff-Van Gieson, Thermo Scientific, MI, USA) or F-actin (ActinGreen™ 488 ReadyProbes, ThermoFisher Scientific, USA) for quantitative analysis. Elastin integrity score was rated by blinded observers and graded on an arbitrary scale of 5 (indicating high quality elastic fiber) to 1 (indicating severe elastin fragmentation). For sequential fluorescence in situ hybridization and immunofluorescence microscopy aortas from human and mice were cryosectioned using OCT standard protocol^70^.

### Statistical analysis

Results are given as mean ± SD. Student’s test was applied to determine the statistical significance of difference between control and treated groups (*p < 0.05, **p < 0.01 and ***p < 0.001). For all experiments at least three replicates were performed. CLIP-seq reads were aligned to human UCSC h19 using standard Illumina library-type. Cufflinks 2.0.2 was run on merged bam files to assemble of transcripts and estimation of FPKM values. FPKM ≤ 1.0 cutoff was used as detection threshold to generate the list of genes. One-way analysis of variance (ANOVA) was used to analyze ultrasound and histology data involving multiple mouse genotypes (95% confidence interval is plotted). *P*-values represent one-way ANOVA followed by Tukey’s honestly significant difference (HSD) post-hoc test. All graphs were produced using GraphPad Prism 7.0.

### Data availability

All relevant data are available from authors. Genomic data from CLIP-seq experiments was deposited to GEO under the access number GSE41607.

## Electronic supplementary material

Supplementary Information

Description of Additional Supplementary Files

Supplementary Data 1

Supplementary Data 2

Supplementary Data 3

Supplementary Data 4

Supplementary Data 5

Supplementary Data 6

Supplementary Data 7
