# Supplementary material for: An HDAC9-MALAT1-BRG1 complex mediates smooth muscle dysfunction in thoracic aortic aneurysm
Source: Nat Commun. 2018 Mar 8;9:1009. doi: 10.1038/s41467-018-03394-7 (PMC5843596; doi:10.1038/s41467-018-03394-7)
Supplement: Supplementary file 1 — Supplementary Information [file 41467_2018_3394_MOESM1_ESM.pdf]

Supplementary Figures

An HDAC9-MALAT1-BRG1 complex mediates smooth muscle dysfunction in thoracic aortic aneurysm

Lino Cardenas et. al.

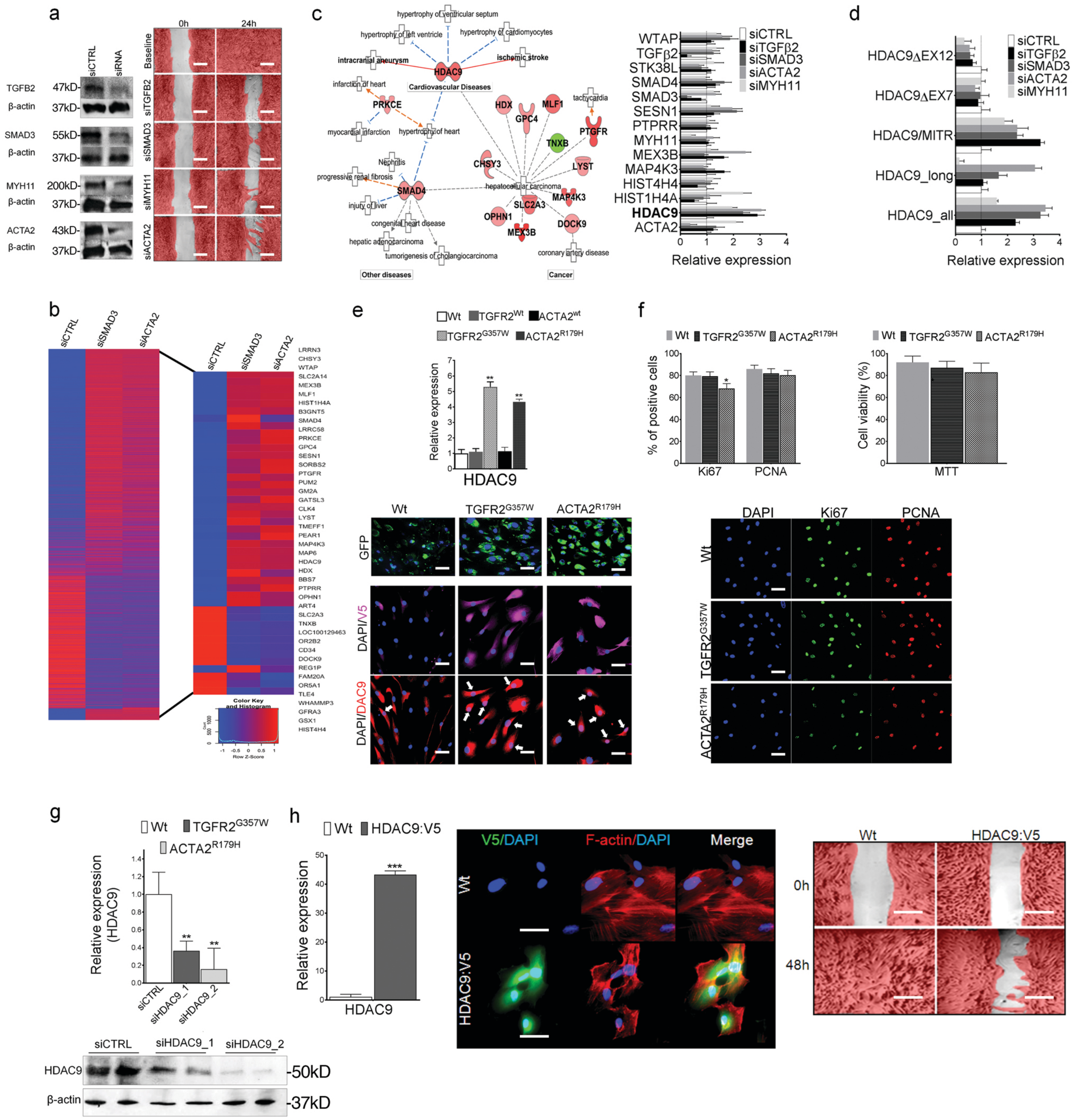

Supplementary Figure 1: (a) siRNA inhibition of genes associated with TAA phenotype and wound healing assay showing impaired features in siRNA treated group compared with siCTRL group after 24 hrs. Bar= 400μM. (b) Heat map of microarrays gene expression in siCTRL, siSMAD3 and siACTA2 treated human VSMCs. Toxicology and cellular pathways listed in Ingenuity Pathway Analysis (IPA) analysis of the 44 commonly dysregulated genes. (c) Gene network representation of highly rated gene-disease network on the 44 genes using Ingenuity Pathway Analysis (IPA) demonstrates strong association of HDAC9 in cardiovascular related diseases. QPCR validation of 14 randomly chosen genes amongst 44 total in siSMAD3, siTGFB2, siACTA2, siMYH11 treated cells compared with siCTRL group. HDAC9 is up-regulated in siRNA treated VSMCs. (d) QPCR analysis of total HDAC9 and 4 HDAC9 isoforms in siRNA treated VSMCs. (e) QPCR analysis of HDAC9 in VSMC transduced with virus particles overexpressing wild-type or aneurysm alleles for TGFR2 and ACTA2 genes ($TGFR2^{Wt}$ or $TGFR2^{G357W}$ and $ACTA2^{Wt}$ or $ACTA2^{R179H}$). Immunofluorescence microscopy showing VSMCs (top row) transduced with empty or $TGFR2^{G357W}$ or $ACTA2^{R179H}$ mutant alleles lentiviral plasmid (pLenti7.3/V5-DEST-EmGFP). Middle row demonstrates similarly transfected VSMCs stained for V5-tagged (magenta) mutant proteins with Anti-V5 antibody. Lower row shows HDAC9 expression (red) identified with anti-HDAC9 antibody. (Student's T-test versus wildtype, *p<0.05, **p<0.01, ***p<0.001) Bar= 10μM. Three experimental replicates are quantified. (f) Proliferation assayed via Ki67 and PCNA staining and viability by MTT assay of human VSMCs transfected with expressing WT, $TGFR2^{G357W}$, or $ACTA2^{R179H}$ alleles. (g) QPCR (top) and immunoblotting (bottom) showing the bioactivity of two set of siRNAs against HDAC9 transcript. (Student's T-test versus wildtype, *p<0.05, **p<0.01, ***p<0.001) Bar= 10μM, Three experimental replicates are quantified. (h) qPCR analysis of HDAC9 transcript in cell transduced with viral particles overexpressing V5-tagged HDAC9 protein. Three experimental replicates are quantified. Middle panel, immunofluorescence microscopy showing nuclear localization of ectopic HDAC9 (green) associated with cytoskeleton dysregulation Bar= 10μM (Red, F-actin). Right panel, over-expression of HDAC9 is sufficient to inhibit VSMC migration. Bar= 400μM. Error bars represent the mean of at least three experimental replicates.

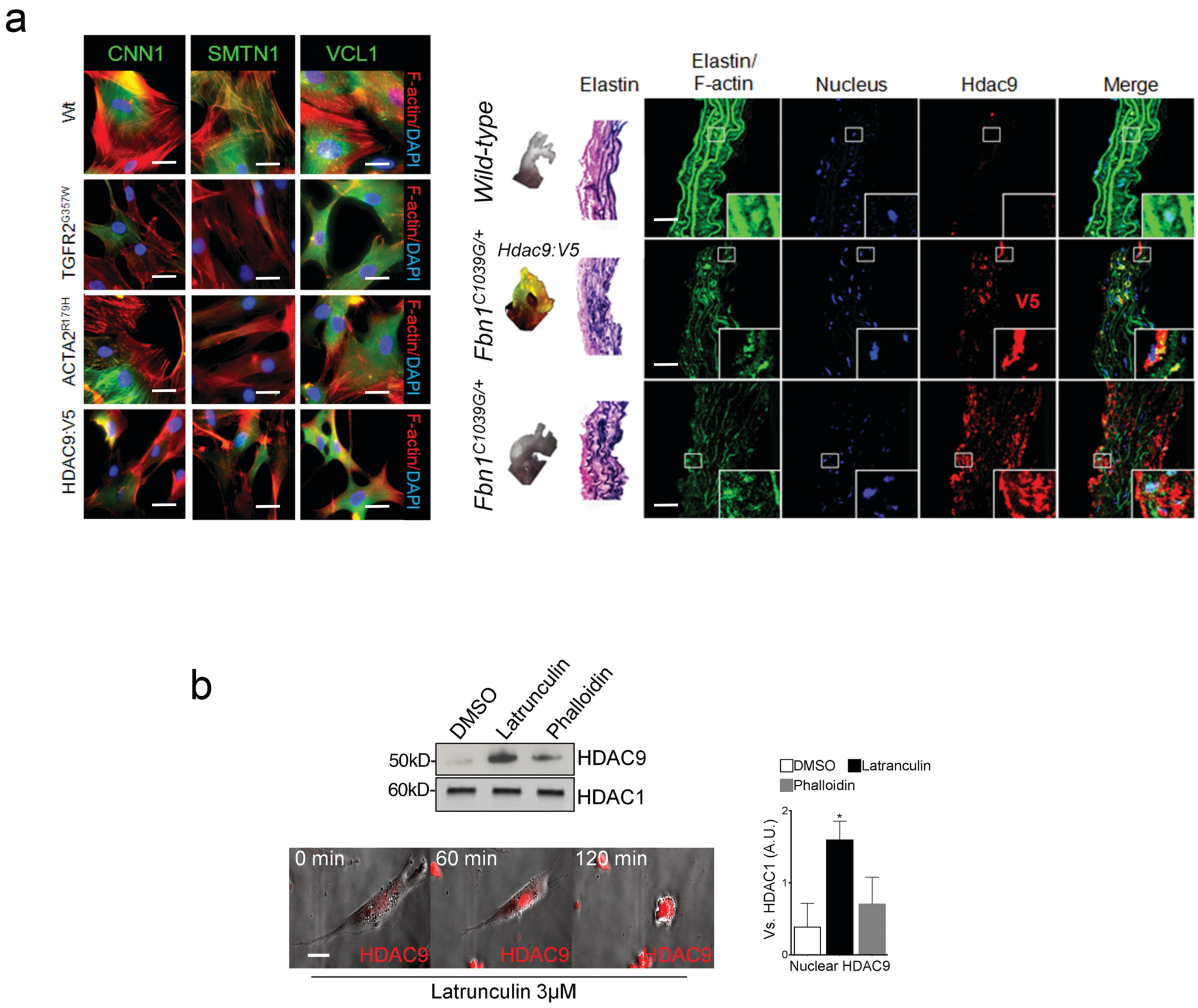

Supplementary Figure 2: (a) Immunofluorescence showing downregulation of contractile proteins in $TGFR2^{G357W}$ or $ACTA2^{R179H}$ expressing cells as well as direct HDAC9 overexpression. Bar= 10μM. Right panel, *ex vivo* overexpression of HDAC9:V5 in wild type aortas shows correlation of HDAC9 ectopic expression (red-V5) with irregular aortic wall architecture (Green, F-actin). Bar= 40μM (b) Exposure of VSMCs to the actin toxins latrunculin (destabilizing) or phalloidin (stabilizing). Upper panel demonstrates HDAC9 western blot analysis of nuclear extracts from latranculin or phalloidin treatment demonstrating increased HDAC9 nuclear ratio over HDAC1. (Student's T-test versus wildtype, $*p<0.05$, $**p<0.01$, $***p<0.001$) Three experimental replicates are quantified. Live cell analysis of RFP-HDAC9 fusion protein when exposed to latrunculin, shows increased HDAC9 nuclear concentration prior to cellular collapse. Bar= 15μM.

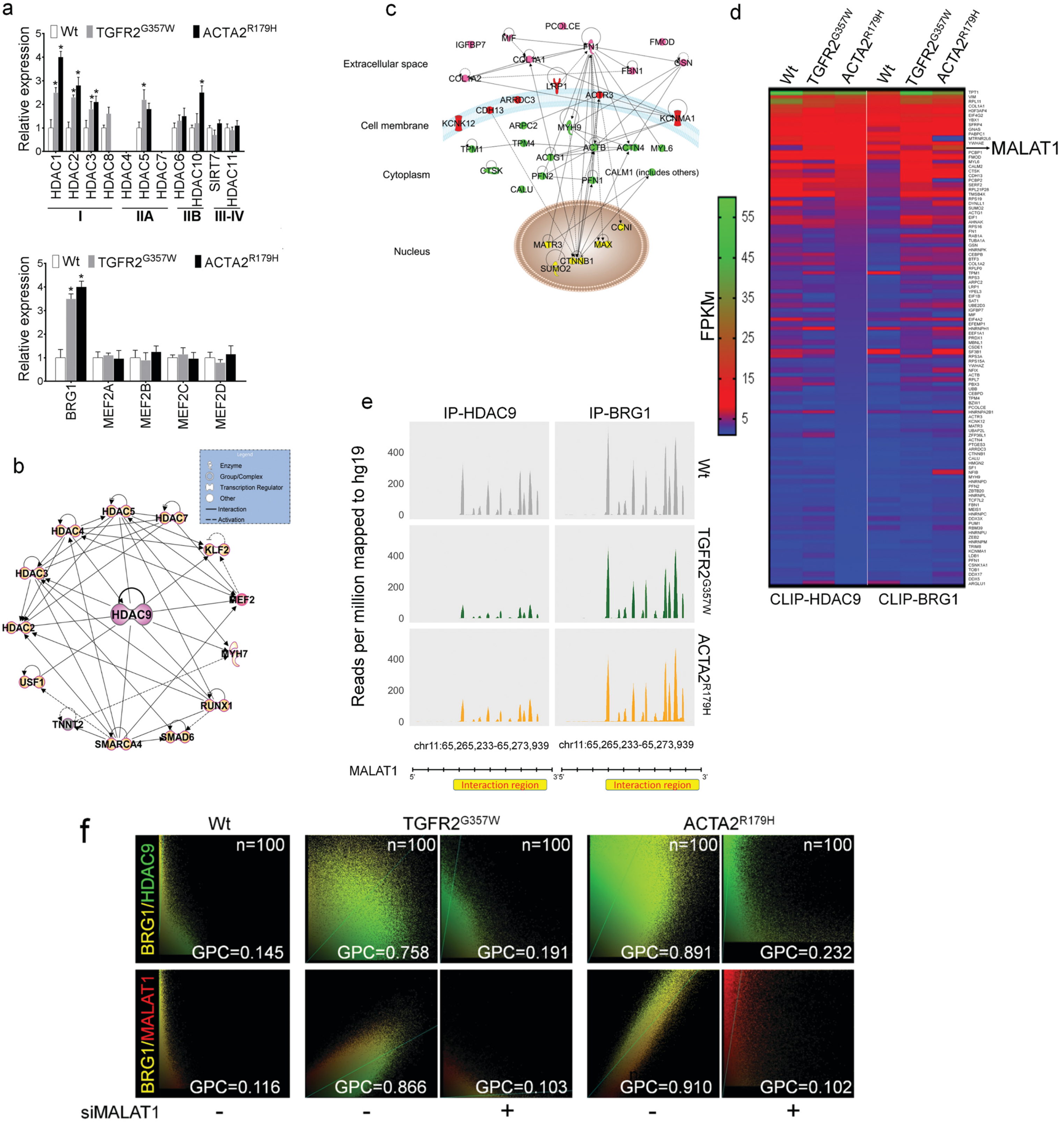

Supplementary Figure 3: (a) QPCR analysis of HDAC isoforms in cells overexpressing TGFR2^{G357W} or ACTA2^{R179H} alleles. Four experimental replicates are shown. (b) Ingenuity Pathway Analysis (IPA) analysis of HDAC9 predicts interaction of HDAC9 with BRG1 (SMARCA4). QPCR analysis of MEF2 isoforms and BRG1 in cells overexpressing TGFR2^{G357W} or ACTA2^{R179H} alleles. (c) Ingenuity Pathway Analysis (IPA) of CLIP-seq identified genes showing multiple cytoskeletal-associated proteins. (d) Heat map of CLIP-seq assay showing 107 transcripts immunoprecipitated with HDAC9 or BRG1 in wild type and/or TGFR2^{G357W} or ACTA2^{R179H} overexpressing cells. (e) Zoom of the human chromosome 11 at MALAT1 locus showing stacked CLIP-Seq reads demonstrating HDAC9 or BRG1 association. Samples obtained from cells expressing vector, TGFR2^{G357W}, or ACTA2^{R179H} alleles are shown. (f) Scarlet plot showing the global pearson colocalization (GPC) of BRG1/HDAC9 or BRG1/MALAT1 overlap in siCTRL and siMALAT treated VSMCs overexpressing TGFR2^{G357W} or ACTA2^{R179H} alleles. (Student's T-test versus wildtype, *$p<0.05$, **$p<0.01$, ***$p<0.001$) Error bars represent the mean of at least three experimental replicates.

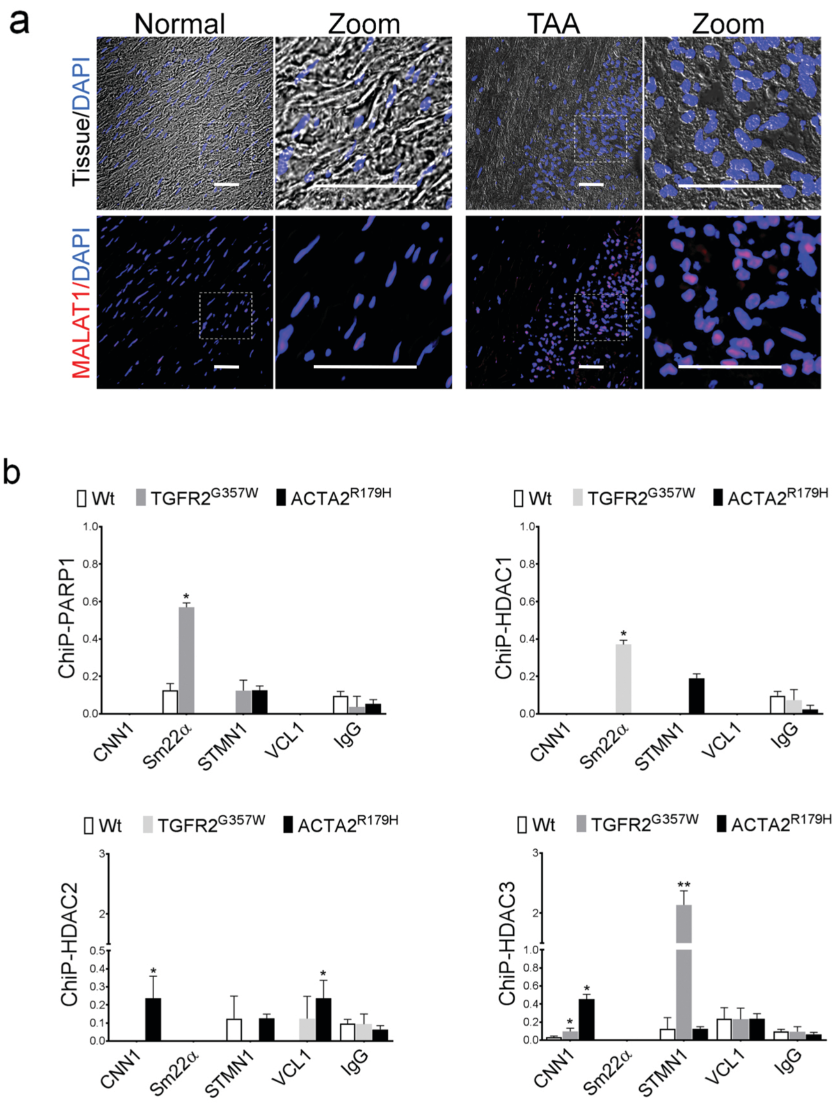

Supplementary Figure 4: (a) Fluorescence in situ hybridization (FISH) demonstrates increased levels of MALAT1 in control and human aneurysm sample. Bar= 50μM (b) Chromatin-immunoprecipitation assays of HDAC1, HDAC2, HDAC3, and PARP1 in VSMCs overexpressing TGFR2^{G357W} or ACTA2^{R179H} alleles demonstrating variable promoter occupancy in contractile genes. (Student's T-test versus wildtype, *p<0.05, **p<0.01, ***p<0.001). Four experimental replicates are shown.

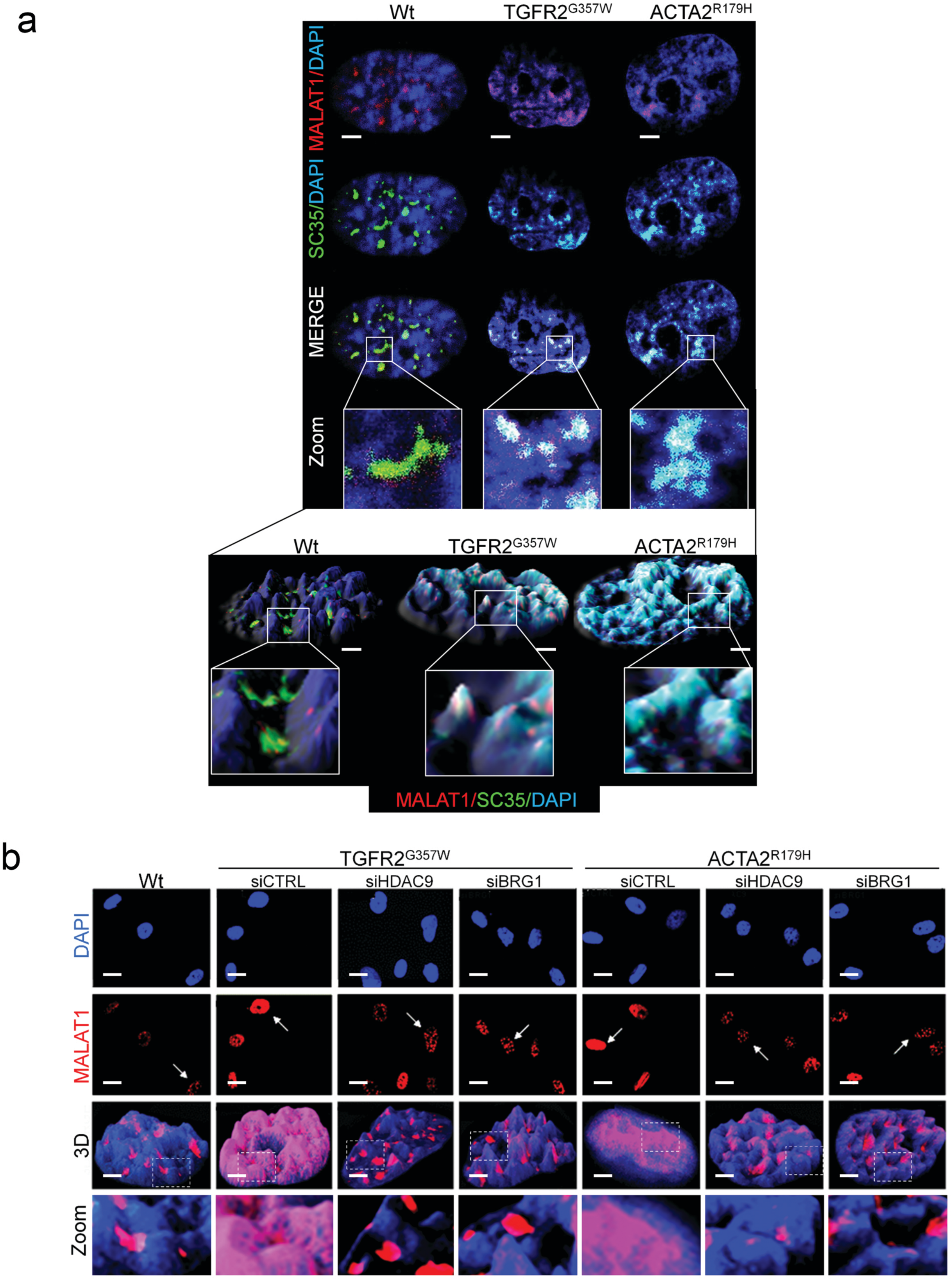

Supplementary Figure 5: (a) VSMCs expressing TGFR2^{G357W} or ACTA2^{R179H} alleles show abnormal relocalization of MALAT1 (red) and SC35 (green), a marker of nuclear speckles, to regions with condensed heterochromatin indicated by DAPI (blue) (Upper Panels, Bar= 2μM). Lower Panels demonstrate 3D surface plot of a single cell to illustrate relationship between SC35, MALAT1 and density of DAPI staining. Bar= 1.5μM (b) Treatment of VSMCs expressing TGFR2^{G357W} or ACTA2^{R179H} alleles with siBRG1 or siHDAC9 relocates MALAT1 (red) to wild type localization pattern. (Upper two rows, Bar= 15μM, third row Bar= 15μM, fourth row 3X zoom of area within dotted line box)

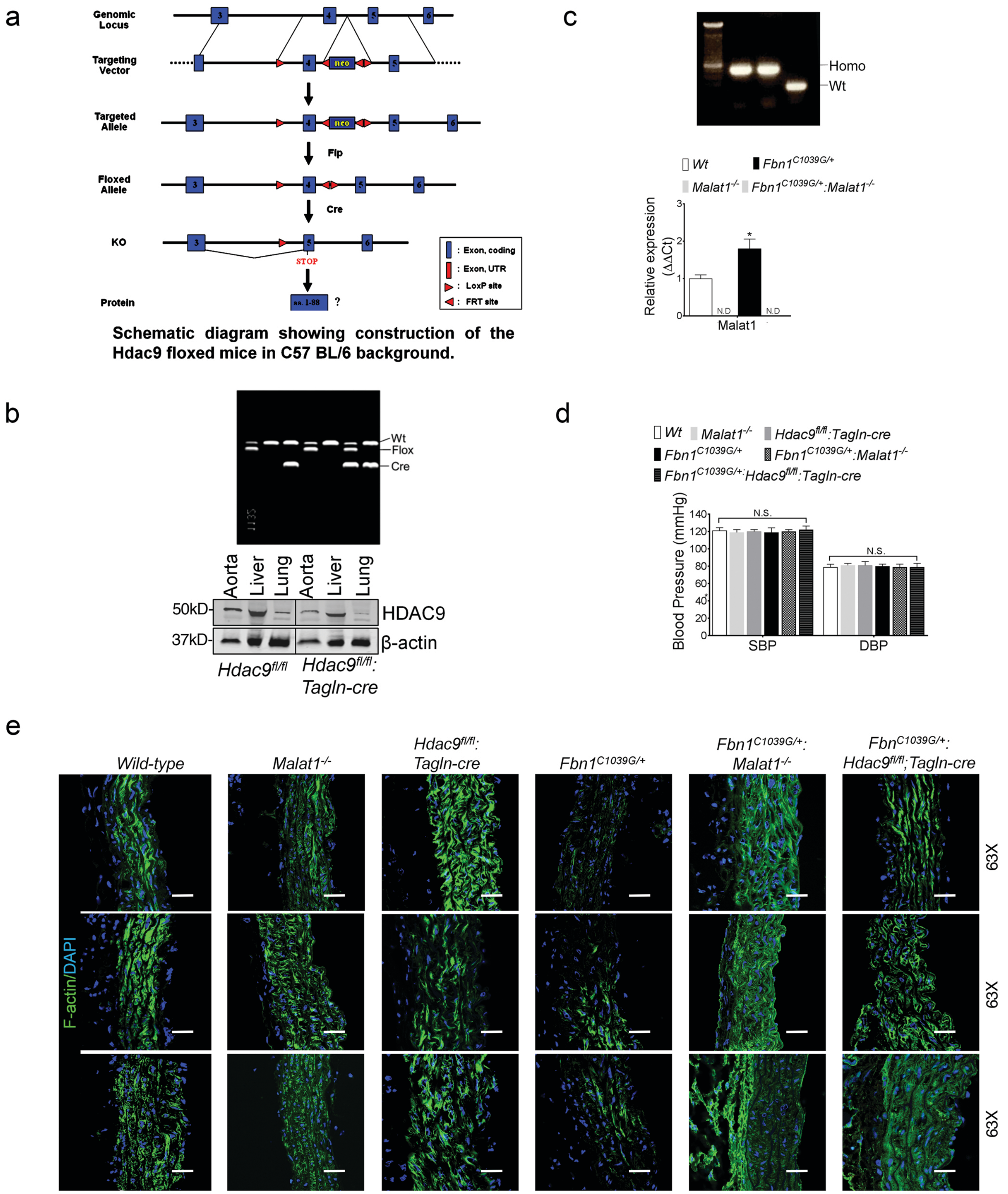

Supplementary Figure 6a-e: (a) Schematic diagram showing construction strategy of Hdac9 floxed mice in C57 BL/6 background. (b) (Upper panel), Genotyping of *Hdac9*$^{fl/+}$ and wild type mice. (Lower panel), western blot of Hdac9 and beta actin from aortic, liver, and lung samples in *Hdac9*$^{fl/fl}$ and *Hdac9*$^{fl/fl}$*:Tagln-cre* mice. (c) Genotyping gel of homozygote *Malat1*$^{-/-}$ and wild type animals (upper panel). Qpcr-based *Malat1* quantification from wildtype, *Fbn1*$^{C1039G/+}$, *Malat1*$^{-/-}$, and *Fbn1*$^{C1039G/+}$*:Malat1*$^{-/-}$ aortas (bottom panel) (Student's T-test versus wildtype, *p<0.05, **p<0.01, ***p<0.001). Three experimental replicates are shown. (d) Blood pressure from wild type, *Malat1*$^{-/-}$, *Hdac9*$^{fl/fl}$, *Fbn1*$^{C1039G/+}$, and *Fbn1*$^{C1039G/+}$*:Malat1*$^{-/-}$, and *Fbn1*$^{C1039G/+}$*:Hdac9*$^{fl/fl}$*:Tagln-cre* mice. (e) Representative staining of F-actin in murine aortas from wild-type, *Malat1*$^{-/-}$, *Hdac9*$^{fl/fl}$*:Tagln-cre*, *Fbn1*$^{C1039G/+}$, *Fbn1*$^{C1039G/+}$*:Malat1*$^{-/-}$, and *Fbn1*$^{C1039G/+}$*:Hdac9*$^{fl/fl}$*:Tagln-cre* mice (Three representative images shown). The aortic adventitia faces left while the aortic lumen is oriented to the right. Bar= 40μM.

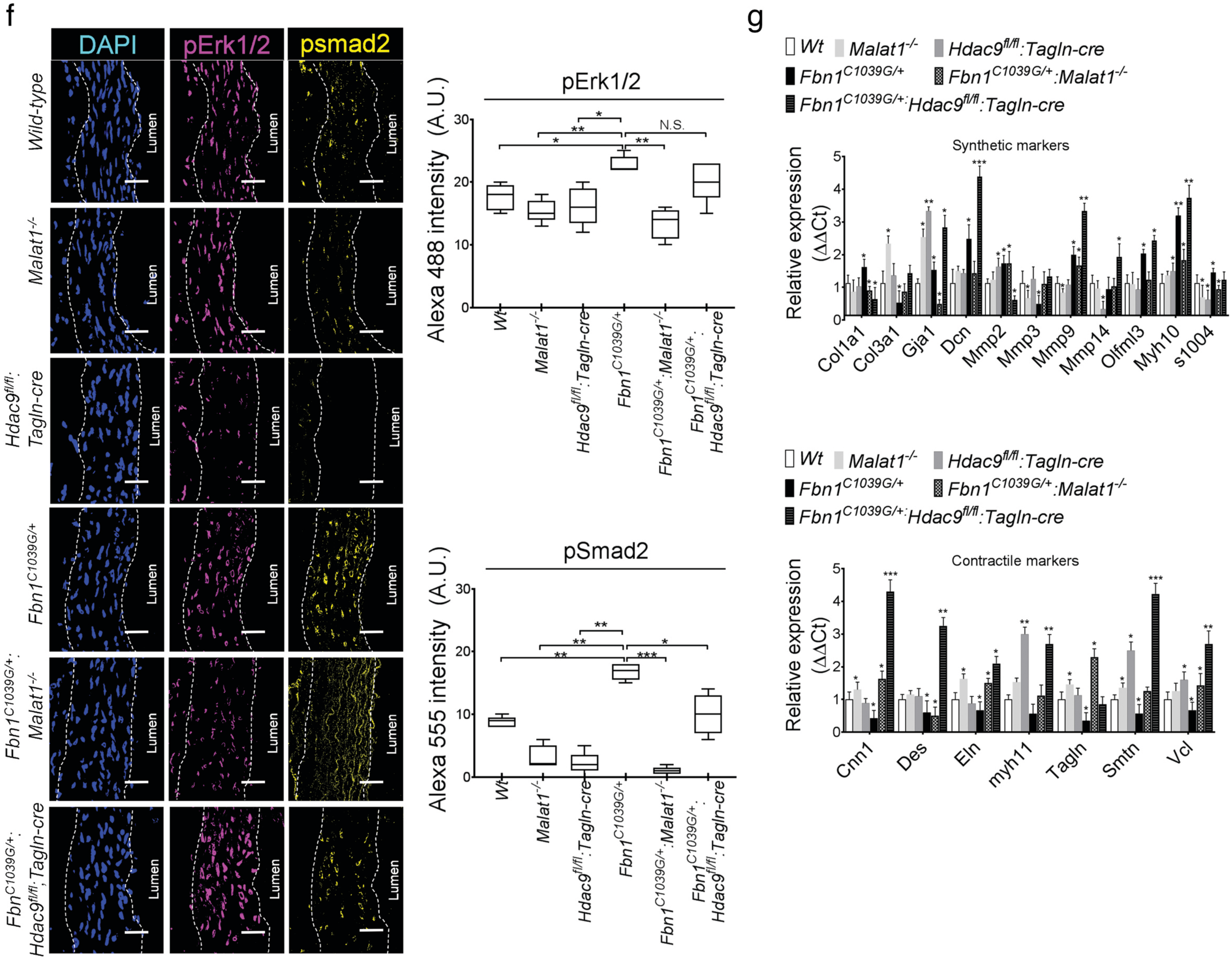

Supplementary Figure 6f&g: (f) Malat1 or Hdac9 deficiency decreases pERK1/2 and pSMAD2 staining in *Fbn1*$^{C1039G/+}$ mice. Immunofluorescence staining of aortic media. Shown are DAPI (blue), pErk1/2 (magenta) and pSmad2 (yellow), in wild-type, *Malat1*$^{-/-}$, *Fbn1*$^{C1039G/+}$, *Fbn1*$^{C1039G/+}$*:Malat1*$^{-/-}$, and *Fbn1*$^{C1039G/+}$:*Hdac9*$^{fl/fl}$*:Tagln-cre* mice. Quantification shown on right. (One-way Anova, *p<0.05, **p<0.01, ***p<0.001). Bar= 40μM. Three photomicrographs per aorta were captured, four animals per genotype. (g) QPCR quantification of synthetic and contractile markers from wild-type, *Malat1*$^{-/-}$, *Hdac9*$^{fl/fl}$*:Tagln-cre*, *Fbn1*$^{C1039G/+}$, *Fbn1*$^{C1039G/+}$*:Malat1*$^{-/-}$, and *Fbn1*$^{C1039G/+}$:*Hdac9*$^{fl/fl}$*:Tagln-cre* mice. (Student's T-test versus wildtype, *p<0.05, **p<0.01, ***p<0.001). Four experimental replicates are shown.

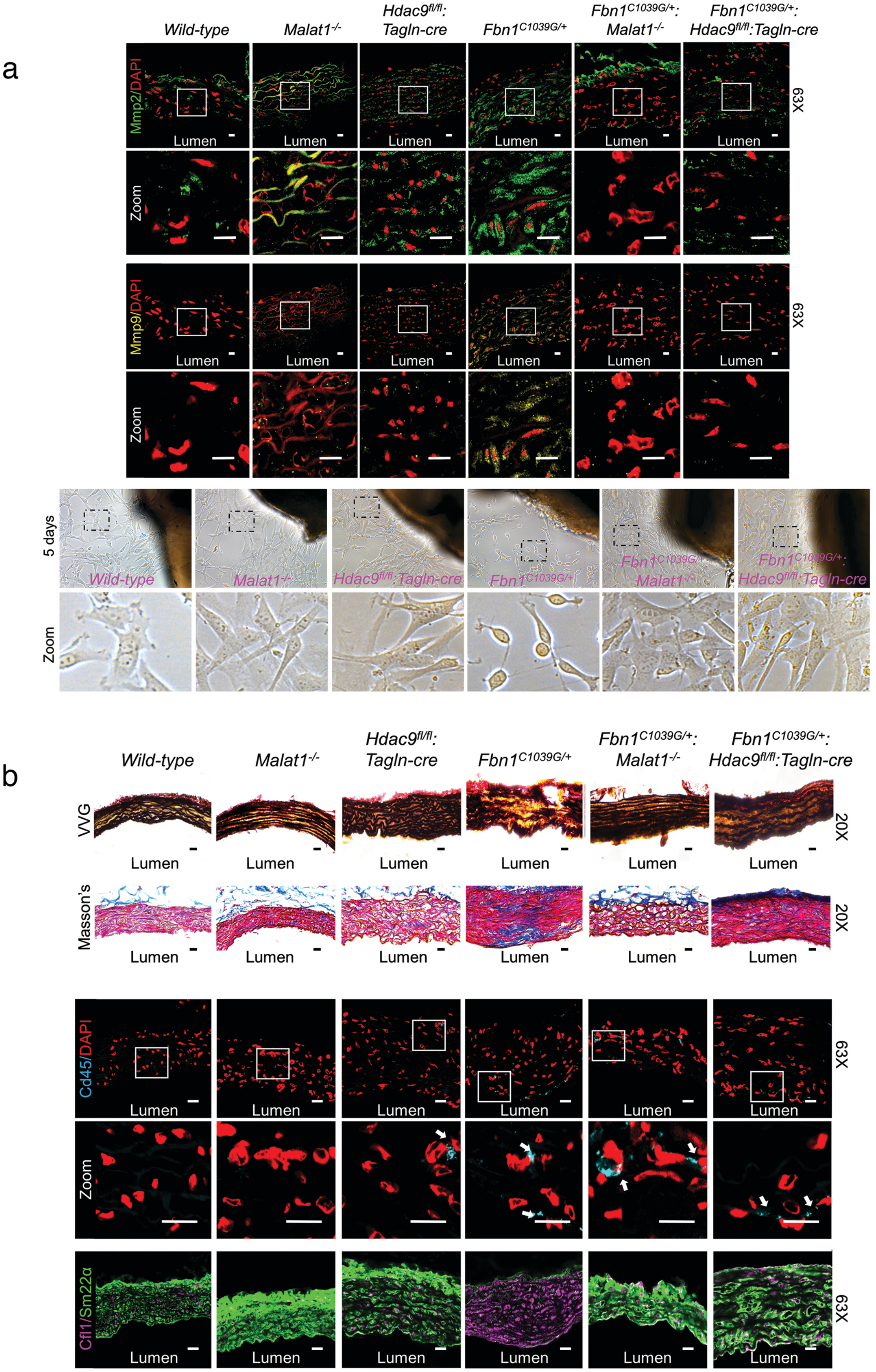

Supplementary Fig. 7 : (a) (Top) Mmp2 and Mmp9 staining in aortic tissue from six month old wild-type, *$Malat1^{-/-}$*, *$Hdac9^{fl/fl}$:Tagln-cre*, *$Fbn1^{C1039G/+}$*, *$Fbn1^{C1039G/+}$:$Malat1^{-/-}$*, and *$Fbn1^{C1039G/+}$:$Hdac9^{fl/fl}$:Tagln-cre* mice. The majority of immunoreactivity resides in the aortic medial VSMCs. (Bottom) Cell migration after 5 days from cultured ascending aortas from wild-type, *$Malat1^{-/-}$*, *$Hdac9^{fl/fl}$:Tagln-cre*, *$Fbn1^{C1039G/+}$*, *$Fbn1^{C1039G/+}$:$Malat1^{-/-}$*, and *$Fbn1^{C1039G/+}$:$Hdac9^{fl/fl}$:Tagln-cre* mice. (b) (Top rows) VVG and Masson's trichrome staining in aortic tissue from six month old wild-type, *$Malat1^{-/-}$*, *$Hdac9^{fl/fl}$:Tagln-cre*, *$Fbn1^{C1039G/+}$*, *$Fbn1^{C1039G/+}$:$Malat1^{-/-}$*, and *$Fbn1^{C1039G/+}$:$Hdac9^{fl/fl}$:Tagln-cre* mice. (Middle rows) CD45-staining of six month old aortic tissue from wild-type, *$Malat1^{-/-}$*, *$Hdac9^{fl/fl}$:Tagln-cre*, *$Fbn1^{C1039G/+}$*, *$Fbn1^{C1039G/+}$:$Malat1^{-/-}$*, and *$Fbn1^{C1039G/+}$:$Hdac9^{fl/fl}$:Tagln-cre* mice demonstrates lack of significant CD45 cellular infiltration. (Bottom row) *Malat1* or *Hdac9* deficiency decreases cofilin expression in *$Fbn1^{C1039G/+}$* aortas. SM22 (green) and Cofilin (magenta) staining in wild-type, *$Malat1^{-/-}$*, *$Hdac9^{fl/fl}$:Tagln-cre*, *$Fbn1^{C1039G/+}$*, *$Fbn1^{C1039G/+}$:$Malat1^{-/-}$*, and *$Fbn1^{C1039G/+}$:$Hdac9^{fl/fl}$:Tagln-cre* mice. Arrows indicate CD45 infiltration. Bar=30μM.

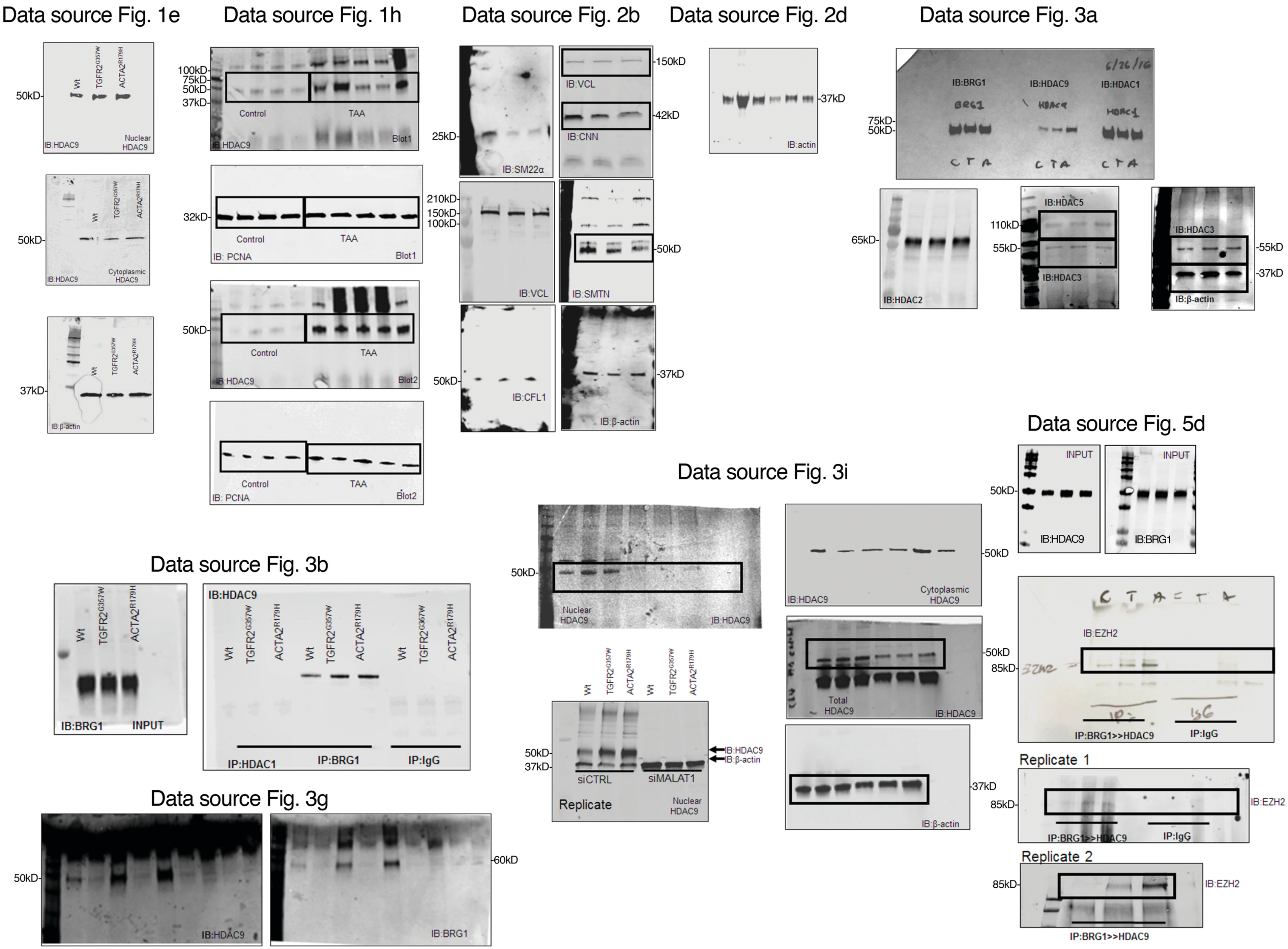

Supplementary Figure 8: Source data for western blots figures 1-5.

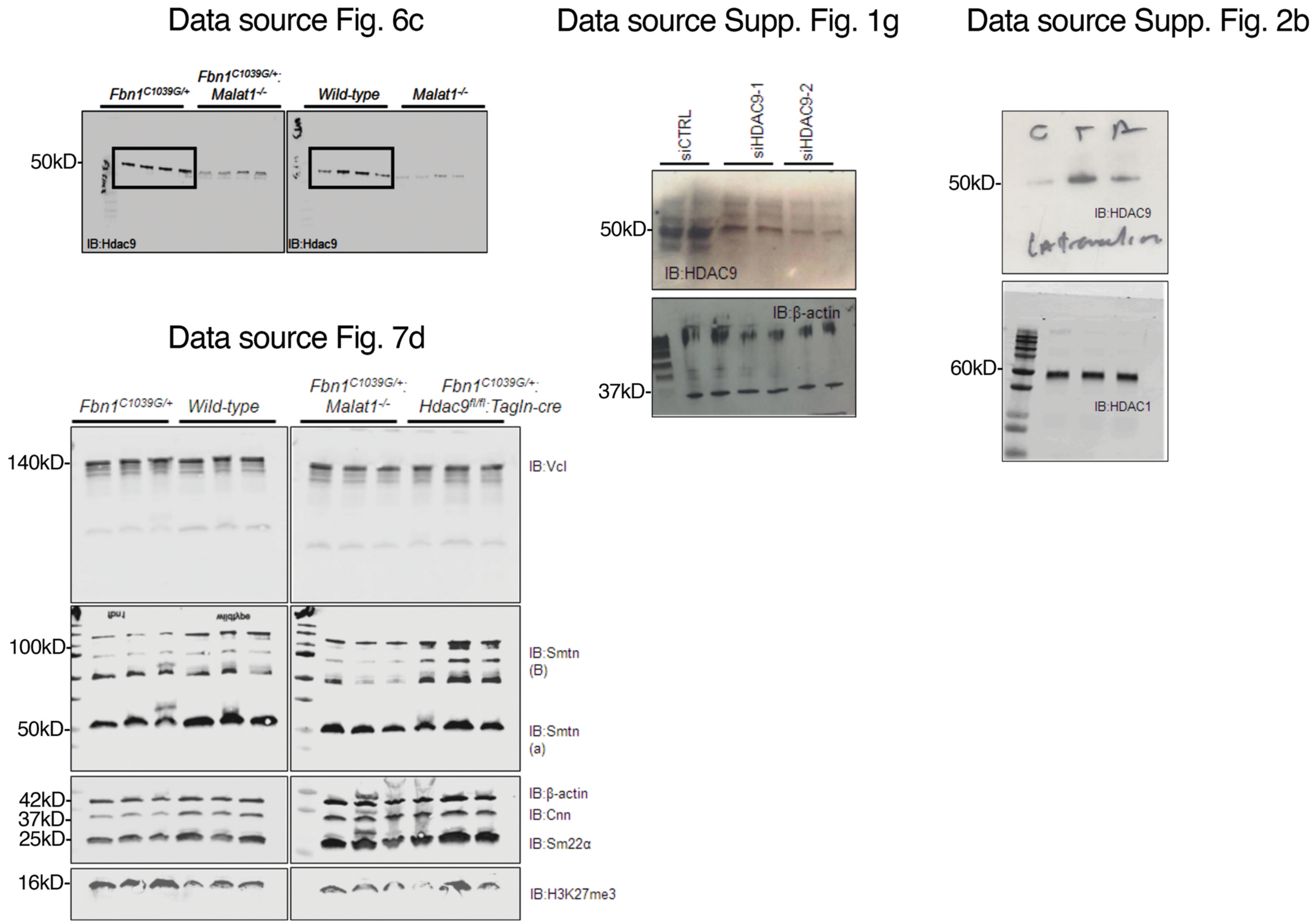

Supplementary Figure 9: Source data for western blots fig 6&7, Supplementary Fig. 1&2.
