# Supplementary material for: An HDAC9-MALAT1-BRG1 complex mediates smooth muscle dysfunction in thoracic aortic aneurysm
Source: Nat Commun. 2018 Mar 8;9:1009. doi: 10.1038/s41467-018-03394-7 (PMC5843596; doi:10.1038/s41467-018-03394-7)
Supplement: Supplementary file 2 — Description of Additional Supplementary Files [file 41467_2018_3394_MOESM2_ESM.pdf]

Description of Additional Supplementary Files

File Name: Supplementary Data 1
Description: Primary data (Fold change and p-values) of genes identified in microarray experiment shown in Figure 1b.

File Name: Supplementary Data 2
Description: Demography of TAA patients from which aneurysm samples were obtained.

File Name: Supplementary Data 3
Description: Genes identified in CLIP-Seq experiment shown in Figure 3c.

File Name: Supplementary Data 4
Description: Ingenuity Pathway analysis of transcripts identified in CLIP-Seq presented in Figure 3c.

File Name: Supplementary Data 5
Description: Prediction of MALAT1 interaction with proteins using RPISeq program.

File Name: Supplementary Data 6
Description: Raw data from mouse echocardiograms plotted in Figure 6.

File Name: Supplementary Data 7
Description: List of materials and reagents used in study including antibodies, primer sequences, probe sequences, and commercial RNAi probes.
